# Synthesis and Characterization of Some New Quinoxalin-2(*1H*)one and 2-Methyl-3*H*-quinazolin-4-one Derivatives Targeting the Onset and Progression of CRC with SAR, Molecular Docking, and ADMET Analyses

**DOI:** 10.3390/molecules26113121

**Published:** 2021-05-23

**Authors:** Nahed N. E. El-Sayed, Taghreed M. Al-Otaibi, Mona Alonazi, Vijay H. Masand, Assem Barakat, Zainab M. Almarhoon, Abir Ben Bacha

**Affiliations:** 1 Egyptian Drug Authority (Formerly: National Organization for Drug Control and Research), 51 Wezaret El-Zerra St., Giza 35521, Egypt; 2 Department of Chemistry, College of Sciences, King Saud University, P.O. Box 2455, Riyadh 11451, Saudi Arabia; chemst222@gmail.com (T.M.A.-O.); ambarakat@ksu.edu.sa (A.B.); zalmarhoon@ksu.edu.sa (Z.M.A.); 3 Biochemistry Department, College of Sciences, King Saud University, P.O. Box 22452, Riyadh 11495, Saudi Arabia; moalonazi@ksu.edu.sa (M.A.); aalghanouchi@ksu.edu.sa (A.B.B.); 4 Department of Chemistry, Vidya Bharati College, Camp, Amravati, Maharashtra 444 602, India; vijaymasand@gmail.com; 5 Department of Chemistry, Faculty of Science, Alexandria University, P.O. Box 426, Ibrahimia, Alexandria 21321, Egypt; 6 Laboratory of Plant Biotechnology Applied to Crop Improvement, Faculty of Science of Sfax, University of Sfax, Sfax 3038, Tunisia

**Keywords:** colorectal cancer, dysbiosis, cyclooxygenase-2, lactate dehydrogenase A, quinazoline, quinoxaline, ADMET

## Abstract

The pathogenesis of colorectal cancer is a multifactorial process. Dysbiosis and the overexpression of COX-2 and LDHA are important effectors in the initiation and development of the disease through chromosomal instability, PGE2 biosynthesis, and induction of the Warburg effect, respectively. Herein, we report the in vitro testing of some new quinoxalinone and quinazolinone Schiff’s bases as: antibacterial, COX-2 and LDHA inhibitors, and anticolorectal agents on HCT-116 and LoVo cells. Moreover, molecular docking and SAR analyses were performed to identify the structural features contributing to the biological activities. Among the synthesized molecules, the most active cytotoxic agent, (**6d**) was also a COX-2 inhibitor. In silico ADMET studies predicted that (**6d**) would have high Caco-2 permeability, and %HIA (99.58%), with low BBB permeability, zero hepatotoxicity, and zero risk of sudden cardiac arrest, or mutagenicity. Further, (**6d**) is not a potential P-gp substrate, instead, it is a possible P-gpI and II inhibitor, therefore, it can prevent or reverse the multidrug resistance of the anticancer drugs. Collectively, (**6d**) can be considered as a promising lead suitable for further optimization to develop anti-CRC agents or glycoproteins inhibitors.

## 1. Introduction

In the Kingdom of Saudi Arabia, colon and rectal cancer (CRC) is the second most common cancer type, constituting 14.4% of all the newly diagnosed cancer cases in 2018 [1]. Although little is known about the exact causes of CRC, however, several modifiable parameters and genetic risk factors have been related to the onset and progression of the disease [2,3].

Concerning initiation of the CRC, dysbiosis associated with the abundance of: *Bacteroides fragilis (B. fragilis)*, *Escherichia coli* (*E. coli*), and *Enterococcus feacalis (E. feacalis)* strains has been shown to contribute to neotumorigenesis [4]. This occurs through diverse mechanisms, such as provoking of proinflammatory cascades, interaction with the host’s immune system, production of cancer-associated metabolites (*N*-nitroso compounds and acetaldehyde), and the release of genotoxins to induce DNA or chromosomal damage or via the production of virulence adherence factors that interfere with the β-catenin signaling and proinflammatory cytokines [5]. Therefore, antibiotics are used as anticancer agents [6].

Moreover, cyclooxygenase isoform-2 (COX-2) and lactate dehydrogenase A (LDHA) are among the enzymes that have been identified to play sophisticated roles in colorectal tumorigenesis.

Despite that, the expression of COX-2 is negligible in normal cells adjacent or distant from the tumors, it is significantly elevated in colorectal neoplastic tissues [7]. The involvement of COX-2 in the early onset [8] and the progression [9] of CRC was established based on randomized trials, which indicated that the administration of CO-X inhibitors, such as nonsteroidal antiinflammatory drugs (NSAIDs), can provide protection from CRC in humans [10]. Moreover, several NSAIDs have been shown to reduce the risk of surgical related metastasis, cancer recurrence in addition to increasing the patients overall survival [11].

Mechanistically, COX-2 is implicated in colorectal carcinogenesis by mediating the conversion of arachidonic acid (AA) to prostaglandins E2 (PGE2). These COX-2 derived bioactive products act to reprogram the tumor microenvironment from antitumor responses to procarcinogenic and immune-evasion responses through promoting angiogenesis, insensitivity to antigrowth signals, uncontrolled tumor cells proliferation, migration, and evasion of apoptosis [12,13]. Consequently, the COX-2/PGE2 pathway is considered an important target in CRC prevention and therapy [14].

In addition to this, during the development of the tumors, the transformed cells switch from mitochondrial respiration to aerobic glycolysis, the ‘‘Warburg effect’’ [15], to obtain their increased need from energy and nutrients. This reprogrammed metabolism is associated with the upregulation of LDHA that acts to diminish pyruvate entry into the tricarboxylic acid cycle (TCA) in the mitochondria, and facilitates its rapid conversion to lactate in the cytoplasm. The produced lactate interferes with the inflammatory and immune responses that regulate cell proliferation, differentiation, migration and death [16]. Moreover, this onco-metabolite is involved in the activation of hypoxia-inducible factor 1-alpha (HIF-1α), which mediates the adaptive alternations responsible for angiogenesis, cell survival, and glucose metabolism [17]. Thus, the suppression of LDHA is one of the proposed mechanisms for anticancer therapy [18].

Quinoxaline and quinazoline and their derivatives are considered as important synthetic targets in anticancer drug discovery [19,20,21,22,23,24]. Quinazoline derivatives have been reported to exhibit cytotoxic activity against different cancer types via various mechanisms, including the inhibition of; epidermal growth factor receptor (EGFR) [25], or kinesin spindle protein (KSP) [26], interfering with: Wnt signaling pathway [27] and phosphatidylinositol-3-kinase (PI3K) [28], in addition to the downregulation of the antiapoptotic proteins Bcl2 and BclxL [29].

Similarly, quinoxaline derivatives have exerted their antiproliferative effects through myriad molecular mechanisms targeting tubulin polymerization, topoisomerase II-DNA, folate metabolism, protein kinases, various receptor tyrosine kinases (RTKs) including vascular epidermal growth factor receptor (VEGFR), epidermal growth factor receptor (EGFR), human epidermal receptor 2 (HER2), and fibroblast growth factor receptors (FGFRs) [30].

Considering the multifactorial nature of CRC and in continuation to our ongoing research targeting this malignancy [29], herein, we report the synthesis and characterization of some new Schiff’s bases incorporating quinoxalin-2(1*H*)one and 2-methyl-3*H*-quinazolin-4-one scaffolds. They were evaluated for antibacterial and enzymatic inhibitory activities against COX-2 and LDHA. Moreover, structure–activity relationship (SAR) analyses were carried out to investigate the effect of different substituents on the enzymatic inhibitory activities.

In addition, the molecules that exhibited promising bioactivity were further screened for anticancer activities against HCT-116 and LoVo colorectal cell lines. Molecular docking analyses of active candidates (**4c**), (**6a**), and (**6d**) were performed in the active sites of their molecular targets: *β*-lactamase PDB: 1a8t, 17beta-HSD5 PDB: 4XVE, and COX−2PDB: 3NT1, respectively, to identify the pharmacophoric centers involved in the important binding interactions. Moreover, all the synthesized compounds were evaluated for drug-like characteristics using the “Lipinski’s rule of five”. Lastly, the absorption, distribution, metabolism, excretion (ADME), and the toxicity properties of all the synthesized compounds were predicted to explore their pharmacokinetic properties

## 2. Results and Discussion

### 2.1. Synthesis and Characterization of the New Quinoxalinone and Quinazolinone Schiff’s Bases

**Scheme 1:**
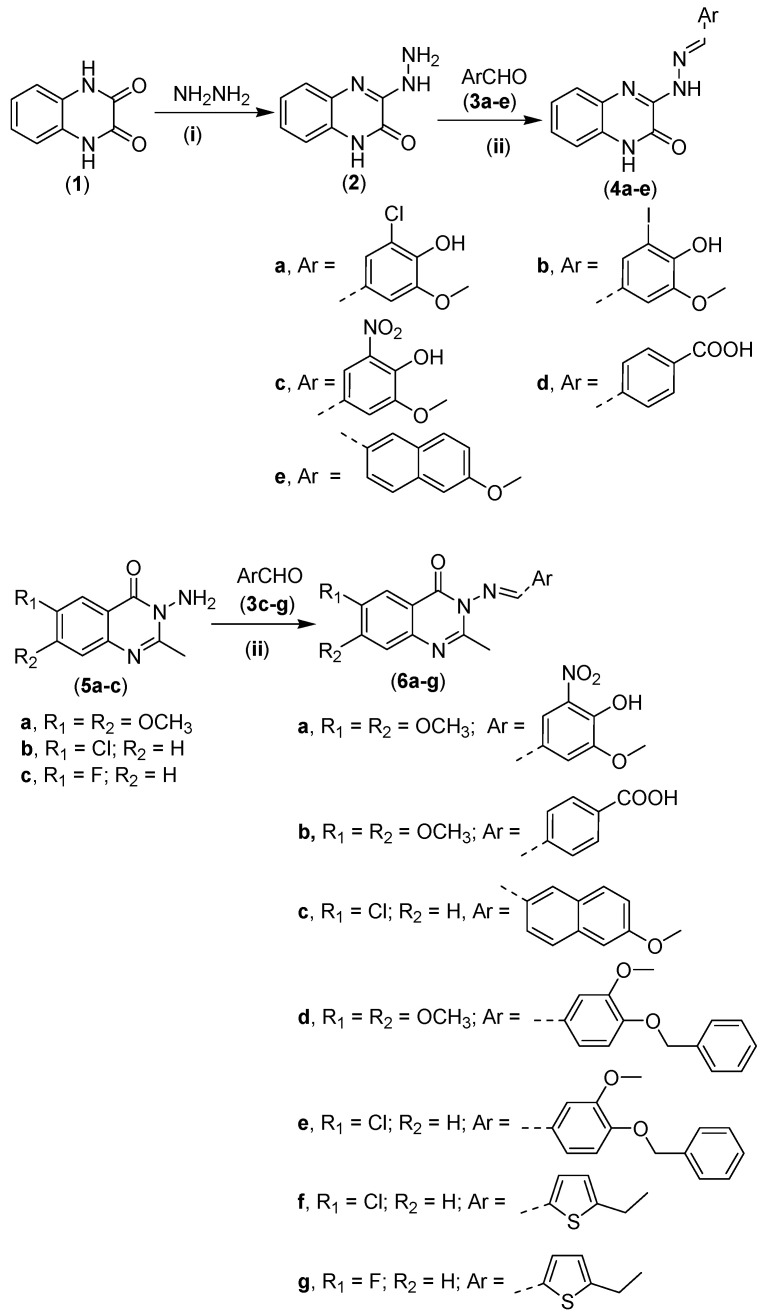
Reagents and conditions: (i) Reflux 6 h; (ii) EtOH/glacial AcOH cat., reflux 10 h.

The syntheses of the precursors, (**1**), (**2**), and (**5a**–**c**) are reported elsewhere [31,32,33,34,35]. The target Schiff’s bases (**4a**–**e**) and (**6a**–**g**) were prepared by condensation of compounds (**2**) and (**5a**–**c**), respectively, with the appropriate aromatic and heteroaromatic aldehydes (**3a**–**g**) in the presence of catalytic amount of glacial acetic acid, as depicted in Scheme 1. 

The structures of the newly synthesized compounds were confirmed by spectroscopic and elemental analyses.

As regard to quinoxalinone derivatives (**4a**–**e**) their FT-IR spectra exhibited the characteristic stretching absorption bands due to 2 × NH, CH-aromatic, C=O, C=N, and C=C groups at ν_max_ = 3320–3304, 3109–3009, 1677–1671, 1620–1615, and 1580–1412 cm^−1^, respectively.

In the ^1^HNMR spectra of these aldimines, it was observed the disappearance of the characteristic signal of the NH_2_ group of the starting 3-hydrazino quinoxalin-2(1*H*)one (**2**), which was detected at *δ_H_* = 4.53 ppm and the appearance of a new one proton signal belonging to the azomethine group (HC=N), resonating at *δ_H_* values ranging from 8.73 to 8.40 ppm. The signals indicative of all the aromatic protons of quinoxalin-2(1*H*)one core and the aryl groups were observed at *δ_H_* = 8.40–7.00 ppm as expected.

Moreover, the ^13^C-NMR spectra revealed the existence of the signals in the ranges of 158.44–151.23, 149.25–146.16, 146.48–144.40, and 155.33–106.66 ppm, which are attributed to CO, C=N, and HC=N, and aromatic-CH and C_q_ of quinoxalin-2(1*H*)one and aryl substituents, respectively.

Considering the spectroscopic data of the Schiff’s bases containing 2-methyl-3*H*-quinazolin-4-one motif (**6a**–**g**), the IR spectra showed the absence of the stretching bands due to the amino groups of the starting amino quinazolinones (**5a**–**c**), which were observed at ν 3303, 3302, and 3311 cm^™^ and the presence of stretching bands at ν 3089–3013, 1676–1663, 1613–1574, and 1482–1419 cm^−1^ indicative of the CH-aromatic, CO, 2 × C=N, and C=C groups, respectively.

In the ^1^HNMR spectra, it was observed the disappearance of the signals due to the NH_2_ groups of the starting amino quinazolinones (**5a**–**c**), which were observed at *δ_H_* = 5.76, 4.92, and 5.82 ppm, respectively, and the appearance of a new one proton singlet signal belonging to the azomethine group resonating at *δ_H_* = 9.04–8.75 ppm. Moreover, the aromatic protons of the quinazolin-4-one ring and the tethered aryl groups were detected at *δ_H_* = 8.23–6.86 ppm.

Additionally, in the ^13^C-NMR spectra, the signals derived from CO, C=N, HC=N, and aromatic-CH and C_q_ of quinazolin-4-one and aryl moieties were observed at *δ_C_* = 167.53–160.22, 159.71–156.82, 157.88–154.20, and 154.54–105.66 ppm, respectively.

Furthermore, the mass spectra displayed the molecular ion peaks corresponding to the molecular formulas. For instance, the mass spectrum of the quinoxalinone derivative (**4e**) revealed the presence of molecular ions at *m/z* (%); [M^+^ + H] 345.12 (2.97) for C_20_H_16_N_4_O_2_, [M^+^] 344.23 (25.39), and the base peak ion at 161.14.

Similarly, the mass spectrum of the quinazolinone derivative (**6d**) indicated the existence of the molecular ion peak [M^+^ + H] at *m/z* 460.18 (25.82%) for C_26_H_25_N_3_O_5_ in addition to the radical cation [M^+^] at *m/z* = 459.33 (100.00%). 

Finally, the results of C,H,N elemental analyses were within the permissible limits for all the new compounds.

### 2.2. Biological Evaluations

#### 2.2.1. Antibacterial Activity

**Figure 1:**
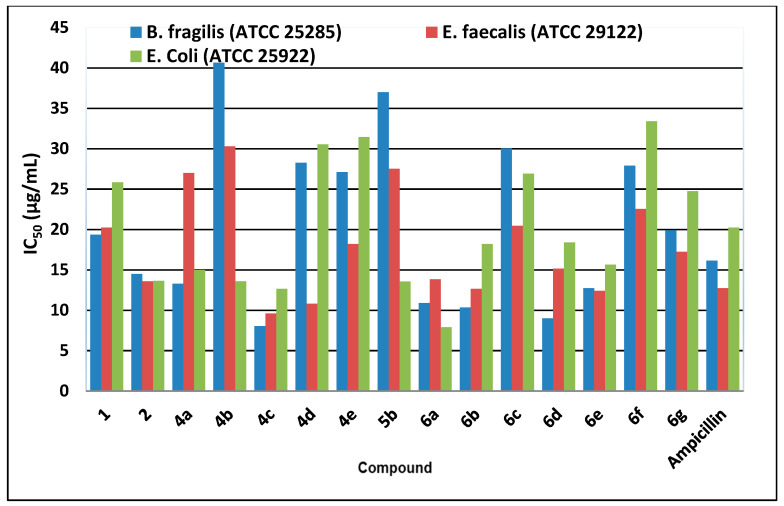
Evaluation of the antibacterial activity of the synthesized quinoxalinone and quinazolinone derivatives by the determination of their IC_50_ values (µg/mL).

The antibacterial evaluation (see Figure 1 and Table S1) was performed by determining the compounds’ IC_50_ values (µg/mL) against some bacterial strains involved in the induction of CRC, namely *E. feacalis*, *B. fragilis,* and *E. coli*. The antibiotic ampicillin was used as the reference drug and its (IC_50_) values against these microbes were determined to be 12.75, 16.15, and 20.25 µg/mL, respectively.

The results showed that the Gram positive strain, *E. faecalis* exhibited the least sensitivity toward the studied compounds with only four derivatives, namely (**4c**), (**4d**), (**6e**), and (**6b**) were capable to exhibit inhibitions at lowered IC_50_ values of 9.60, 10.80, 12.40, and 12.65 µg/mL, respectively compared to ampicillin.

Conversely, the gram negative bacterial strains, *B. fragilis* and *E. coli* were more sensitive to the tested compounds. Thus, seven derivatives, namely (**4c**), (**6d**), (**6b**), (**6a**), (**6e**), (**4a**), and (**2**) and nine compounds, namely (**6a**), (**4c**), (**5b**), (**4b**), (**2**), (**4a**), (**6e**), (**6b**), and (**6d**), exhibited reduced IC_50_ values than that of ampicillin, ranging from 8.05 to 14.50 µg/mL and 7.90 to 18.40 µg/mL against these pathogens, respectively.

As a consequence, compounds (**4c**), (**6b**), and (**6e**) can be recognized as promising broad-spectrum antibacterial candidates against the specified stains. Additionally, due to the connections between bacterial dysbiosis and the induction of CRC, these antibacterial agents may provide protection against CRC.

#### 2.2.2. Enzymatic Inhibitory Assays

##### COX-2 Inhibitory As

**Figure 2:**
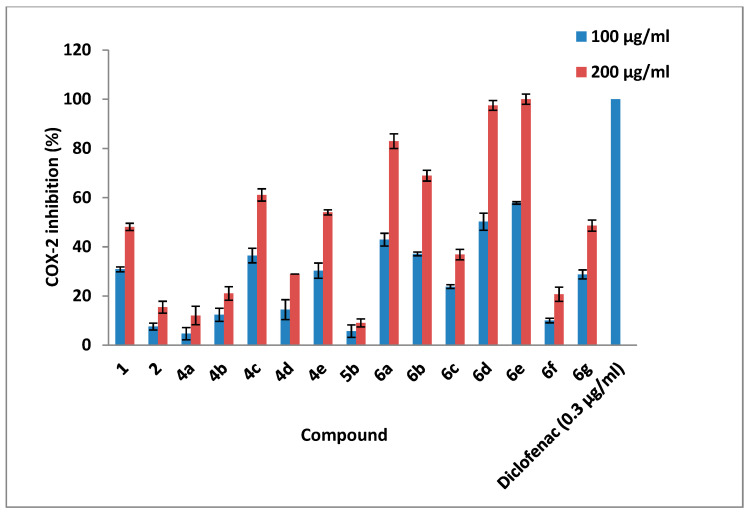
Evaluation of the COX-2 inhibitory properties of the synthesized quinoxalinone and quinazolinone derivatives by the determination of the mean inhibition efficiency at 100 and 200 µg/mL concentrations.

The results of the in vitro assessment of the COX-2 inhibitory activities (see Figure 2 and Table S2) at 100 µg/mL, showed that only compounds (**6e**) and (**6d**) exhibited moderate inhibition efficiencies of 57.85% and 50.20%, respectively. However, the remaining compounds displayed weak inhibition efficiencies ranging from 42.90% to 4.65% compared with 100.00% inhibition efficiency exhibited by the positive control used (diclofenac 0.3 µg/mL).

Contrarily, at 200 µg/mL, the studied compounds demonstrated improved inhibition efficiencies ranging from 100.00% to 9.05%. The most active COX-2 inhibitors were 2-methyl-3*H*-quinazolin-4-ones (**6e**, 100.00%), (**6d**, 97.45%), and (**6a**, 82.95%), whereas, the rest of the studied compounds exerted moderate to poor inhibition efficiency ranging from 68.95% to 9.05%.

Therefore, the mean IC_50_ values (µg/mL) for these active candidates were calculated to be 96.19 ± 5.393582 (**6e**), 99.02 ± 5.088962 (**6d**), and 121.55 ± 1.410302 (**6a**), compared to 0.53 ± 0.042426 produced by diclofenac.

These results are of great impact based on previous mice models experimental studies, which highlighted the relations between the overexpression of COX-2 and the occurrence of CRC. This study showed that COX-2 is responsible for the development of the *APC* mutation-induced intestinal adenoma and the production of the VEGF [31]. Moreover, in another study the antitumor effects of diclofenac; the COX-2 inhibitor was attributed to its ability to induce apoptosis by inactivation of the PI3K and Wnt signaling pathways, which protect the neoplastic colon cells from apoptosis [32].

Consequently, COX-2 inhibitors, (**6e**), (**6d**), and (**6a**) may be considered as promising candidates that can be further optimized to develop new potent chemopreventive or therapeutic agents against colorectal tumorigenesis [33].

SAR studies for COX-2 inhibitory potency revealed that the highest activity was observed with Schiff’s bases (**6e**), (**6d**), (**6a**), and (**6b**) having the quinazolinone core. Within this set, although compounds (**6d**), (**6a**), and (**6b**) have the same type of the substituents (6 and 7-OCH_3_ groups), they displayed different inhibitory efficiencies ranging from 100.00% to 68.95%.

Conversely, compounds (**6e**, having 6−Cl atom) and (**6d**, having 6- and 7-OCH_3_ groups), which possess the 4-benzyloxy-3-methoxy phenyl moiety displayed relatively comparable inhibitory efficiencies of 100.00% and 97.45%, respectively.

However, the combination of strong electron donating groups (3-OCH_3_ and 4-OH) with a strong electron withdrawing substituent (5-NO_2_) as in compound (**6a**), reduced the efficiency to 82.95%.

Moreover, the substitution of the “4-benzyloxy-3-methoxyphenyl group” on the azomethine linker by the “5-ethyl-thiophen-2-yl” moiety diminished the inhibition efficiency as shown in compounds (**6e**, 100.00%) and (**6f**, 20.70%).

Lastly, compound (**5b**), which possesses the 6-Cl-quinazolinone core (same as compound **6e**) and lacks the azomethine and aryl groups, displayed a very poor inhibitory efficiency with value of 9.05%.

All of these observations suggest that the difference in the anti-COX-2 activity among quinazolinone derivatives is mainly dependent on the presence of the azomethine linker and type of the substituent(s) present on the phenyl groups. This is further confirmed by the molecular docking studies.

Similarly, among the quinoxalinone Schiff’s bases, the most active derivatives were (**4c**, 61.10%, having 4-OH,3-OCH_3_,5-NO_2_-phenyl) and (**4e**, 54.00%, having 6-OCH_3_-2-naphthyl), which possess aryl groups having electron donating groups, while the least active derivative was compound (**4d**, 28.90%) bearing the 4-COOH-phenyl moiety. The latter compound was even weaker than compound (**1**, 48.10%), which lacks the azomethine and aryl groups. Although, compounds (**4b**) and (**4a**) possess 4*-* and 5-electron donating substituents (OH and OCH_3_), they exhibited low inhibition efficiencies (21.05% and 12.05%), which may be attributed to the presence of halogen substituents; 3-I and3-Cl, respectively.

Considering all these data together, the most active COX-2 inhibitors among the synthesized derivatives are the Schiff’s bases having a quinazolinone scaffold. Moreover, the inhibition efficiency in both of quinazolinone and quinoxalinone derivatives is enhanced by the introduction of electron donating groups at 3*-* and 4- positions of the aryl moiety and it is reduced by insertion of halogen substituents.

##### LDHA Inhibitory Assay

**Figure 3:**
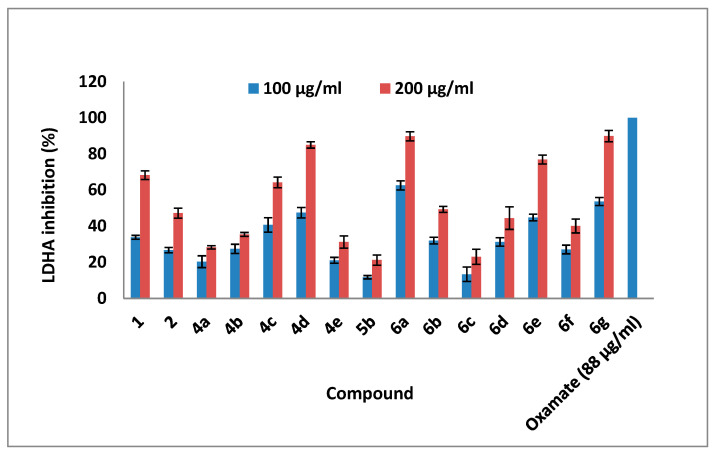
Evaluation of the LDHA inhibitory properties of the synthesized quinoxalinone and quinazolinone derivatives by the determination of the mean inhibition efficiency at 100 and 200 µg/mL concentrations.

The results of the in vitro LDHA inhibitory activities shown in Figure 3 and Table S3 indicated that only compounds (**6a** and **6g**) exhibited inhibition efficiencies higher than 50.00%, i.e., 62.55% and 53.60%, respectively, at 100 µg/mL, compared to 100.00% inhibition efficiency of the reference LDHA inhibitor (oxamate 88 µg/mL). However, at the concentrations of 200 µg/mL, six compounds, namely (**6g**), (**6a**), (**4d**), (**6e**), (**1**), and (**4c**) exhibited improved inhibition efficiencies of 89.85, 89.70, 84.95, 76.85, 68.20, and 64.20%, respectively. The remaining compounds demonstrated weak inhibition efficiencies ranging from 49.25% to 21.20%.

Considering these results, the mean IC_50_ values (µg/mL) of the promising LDHA inhibitors (**6g**), (**6a**), and (**4d**), were calculated to be 105.2901 ± 8.49449, 102.7094 ± 3.922087 and 111.6008 ± 8.648674, respectively compared to 15.6 ± 0.848528 µg/mL exhibited by oxamate.

The SAR investigation results of the LDHA suppression indicated that the most active inhibitors were two quinazolinone derivatives (**6g**, 89.85%) and (**6a**, 89.70), and one quinoxalinone molecule (**4d**, 84.95%), which possess strong electron withdrawing atoms or groups on the heterocyclic core or the phenyl ring.

Among the Schiff bases having quinazolinone scaffold as the central core, it was observed that the inhibition efficiency was enhanced by introducing strong electron withdrawing substituents that can act as H-bond acceptor such as 7-F in (**6g**) and 5-NO_2_ in (**6a**). However, introducing a COOH group (capable of H-bond donation and accepting) was found to produce different effects depending on the type of the heterocyclic core. Thus, unlike the quinoxalinone derivative (**4d**) the inhibition efficiency was relatively high (84.95%), quinazolinone (**6b**) exhibited moderate inhibition efficiency (49.25%).

Moreover, comparing derivatives (**6g**, 89.85%) and (**6f**, 40.05%), both of which possess the 5-ethyl-thiophen-2-yl moiety on the azomethine linker, it was observed that the substitution of a 7-F atom by a 7-Cl decreased the inhibition efficiency by 2.2 fold, which indicate the importance of F atom.

For quinazolines (**6d**) and (**6e**), which have the same aryl group, it was observed that replacement of 6,7-di-OCH_3_ by 7-Cl in the quinazolinone core, improved the inhibition efficiency by 1.7 fold.

Besides, among the 7-chloroquinazolinone derivatives (**5b**, **6c**, **6e**, and **6f**) it was found that the highest inhibition efficiency was obtained when the azomethine group bear an aryl group possessing strong electron donating groups capable of H-bond formation as in (**6e**, having 4-benzyloxy and 3-methoxyphenyl, 76.85%), which was more potent than (**6f**, having 5-ethyl-thiophen-2-yl, 40.05%) and (**6c**, having 6-methoxy-2-naphthyl group, 23%) and (**5b**, lacking azomethine and phenyl groups, 21.20%) by 1.9, 3.3, and 3.6 fold, respectively.

Similarly, for quinoxalinone derivatives, it was observed that the inhibition efficiency was increased by the introduction of strong electron withdrawing substituents on the aryl group or on the main core. Thus compounds, (**4d**, with *p*-COOH), (**1**, with amidic CO), and (**4c**, with *m*-NO_2_), displayed the highest inhibition efficiencies of 84.95%, 68.00%, and 64.20%, respectively. In contrast, the remaining compounds: (**2**, having -NH-NH_2_ moiety); (**4b**, with 4-OH-3-I-5-OMe-phenyl); (**4e**, with 6-methoxyl-2-naphthyl); (**4a**, with 3-Cl-4-OH-5-OMe-phenyl); which lack such substituents, demonstrated weak inhibition efficiencies of 47.15%, 35.40%, 31.20%, and 28.25%, respectively.

Moreover, comparing quinoxalindione (**2**) with Schiff’s bases (**4a**–**c** and **4e**) indicated that the effect of the amidic-carbonyl group of the main core outweigh the effect of azomethine linker and the tethered aryl groups that lack the electron withdrawing substituents.

Based on these data, it can be suggested that an improvement in the LDHA inhibition by quinoxalinone Schiff bases can be achieved by introducing NO_2_ and COOH groups on the aryl group.

For quinazolinone Schiff bases there is no specific trend, however, F and NO_2_ substituents have a positive impact.

#### 2.2.3. Cytotoxicity Studies

The best-in-class compounds, including (**4c**), (**4d**), (**6a**), (**6b**), (**6d**), (**6e**), and (**6g**), which were recognized as antimicrobial and enzymatic inhibitors, were further examined for their cytotoxic effects on LoVo and HCT-116 cells of human colon cancer. This was carried out by determining the residual percentages of viable cells after being treated with the compounds at different concentrations (50, 100, 200, and 400 µg/mL), using triton X-100 (at concentration of 0.1%) in the assay medium and the assay as the positive and negative controls, respectively.

**Figure 4:**
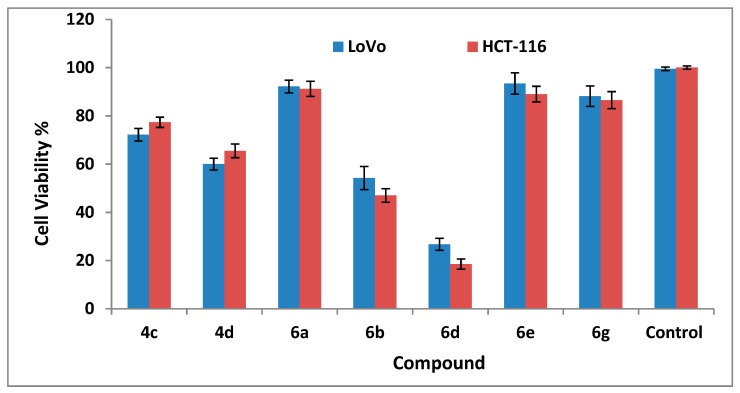
Cytotoxic effects of some selected bioactive Schiff’s bases on LoVo and HCT-116 colorectal cells.

It was found that the percentages of viable cells were reduced over the range of 50–200 µg/mL and they remained constant, at the upper tested concentration (400 µg/mL). Thus, the strongest cytotoxic effects were displayed by the studied compounds at 200 µg/mL, as shown in Figure 4 and Table S4. Among the tested compounds, anti-COX-2 quinazolinone (**6d**) was the most active candidate, as it displayed the lowest percent of the viable cells: 26.75% and 18.50% against LoVo and HCT-116 cells, respectively, compared to 99.50% and 100.00% viability expressed by the negative controls. Thus, the IC_50_ values for this compound were determined to be 127.5 and 100.0 µg/mL on LoVo and HCT-116 cells, respectively.

### 2.3. Molecular Docking Investigations Against Bacterial Targets

#### 2.3.1. Docking Validation

Molecular docking provides significant information about structural features that govern the interactions with the amino acid residues of an enzyme, therefore appropriate validation of docking protocol is very important. A good strategy to achieve this goal is to redock the native ligand in the active site of the enzyme. It is rational to consider that if the docking protocol is correct then there will be high similarity apropos to conformation, position, types of interactions, etc., between the redock pose and X-ray determined pose. The same approach was used in the present work to validate the docking protocol. For COX-2, *E. coli* and *B. fragilis*, the respective native ligands **NPS**, **WDS**, and **061** in the active site of enzymes were re-docked, then a comparison was made between redock pose and X-ray determined structure.

**Figure 5:**
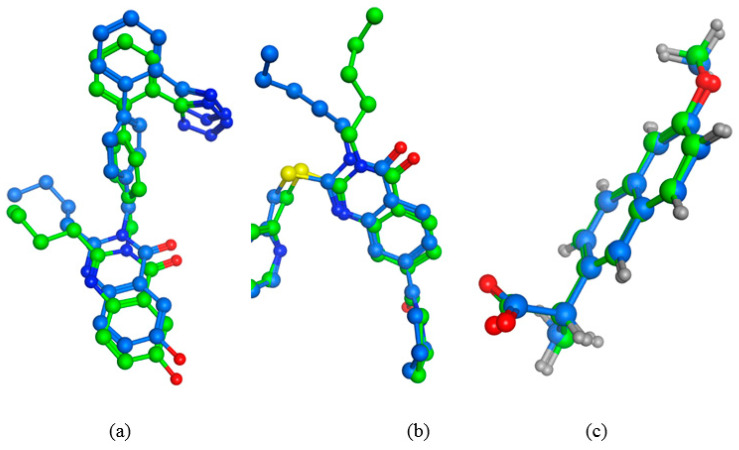
Comparison of X-ray determined structure with docking pose of native ligand (**a**) for *B. fragilis*: PDB 1A8T (blue: X-ray pose, green; docking pose for **061**); (**b**) for *E. coli*: PDB 4XVE (green: X-ray pose, blue: docking pose for **WDS**); (**c**) for COX-2: PDB 3NT1 (blue: X-ray pose, green: dock pose for **NPS**).

In general, there was a high conformational and position similarity with the X-ray resolved structure and the docking pose of the native ligand inside the active site of the respective enzyme. This clearly vindicates that the docking protocol is acceptable. In the case of *B. fragilis*, the docking pose and X-ray structure of native ligand **061** have a difference with respect to the orientation of the *n*–butyl chain linked with the quinazoline ring. Likewise, for *E. coli*, the docking and X-ray determined structures of **WDS** have a difference with respect to *n*-pentyl chain attached to the quinazoline ring. A plausible reason for this dissimilarity could be the high conformational flexibility associated with the carbon chain and availability of enough vacant space inside the active site of the enzyme. While for COX-2, the docking pose and X-ray structure of native ligand **NPS** have high similarity. From Figure 5, it is reasonable to consider that the docking protocol is successful in regenerating an acceptable docking pose, which has a high similarity with X-ray determined structure, thereby validating the docking protocol.

#### 2.3.2. Docking of Compound (**4c**) in the Active Site of Metallo-β-lactamase (*B. fragilis*)

**Figure 6:**
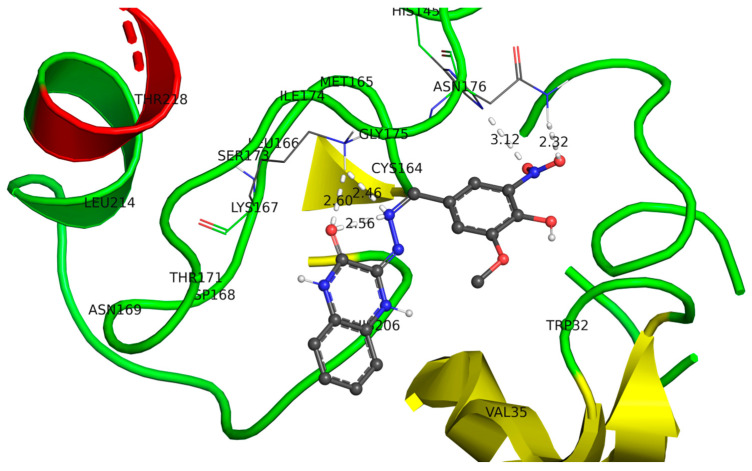
Docking pose of molecule (**4c**) in the active site of metallo-*β*-lactamase pdb: 1a8t.

Many isolates of *Bacteroides fragilis* had developed resistance to *β*-lactam/carbapenem antibiotics because of overexpression of a group of metallo-beta-lactamase enzymes, which can hydrolyze the β-lactam ring of the drug to eliminate its antibacterial properties [34]. Therefore, the most active anti-*B. fragilis* candidate (**4c**) was docked in the active site of metallo-beta-lactamase-class B (pdb: 1a8t) enzyme to investigate the binding interactions between them (Figure 6 and Figure S1).

As shown in the 3D representation of the enzyme–substrate interactions, the molecule has adopted a “J” shape inside the active site of metallo-*β*-lactamase. It exhibited hydrophobic, mild polar, and H-bond interactions with the residues of the active site. The oxygen atom, which is part of the quinoxalinone ring, is involved in the H-bonding with the Gly175 residue at a distance of approximately 2.6 Å. Furthermore, the nitrogen atom of the linker =N-N= group was involved in the H-bonding with the Gly175 residue at a distance of approximately 2.46 Å, which reflects its importance as a linker. Similarly, the nitro substituent attached to the phenyl side group enhanced H-bonding through its interaction with Asn176 (distance 3.12 and 2.32 Å). These interactions can confirm the importance of the nitro substituent and hydrazinoquinoxalinone core for inhibition of this enzyme. Accordingly, the presence of highly polar groups that can form H-bonds on the aryl ring should be retained to have good interactions with the enzyme.

#### 2.3.3. Docking of (**6a**) in the Active Site of 17beta-HSD5 (*E. coil*)

The enzyme, 17-beta-hydroxysteroid dehydrogenase type 5 (17beta-HSD5, PDB code: 4XVE), was chosen as the molecular target to explain the antibacterial potential of compound (**6a**) against *E. coil*. 

Generally, 17beta-HSD5 catalyzes either the oxidation of the hydroxyl group or the reduction of the ketone group at steroid positions C3 and C17. Despite that *E. coil* cannot synthesize steroidal compounds, it has enzymes capable of modifying and/or completely degrading the exogenous steroids, such as the hormones and bile acids of the host, or even glucocorticosteroid (GC) drugs for their own benefits. GC drugs such methylprednisolone, dexamethasone, and flumethasone are commonly administrated in the cases of severe *E. coli* infections to treat the inflammations, or local and systematic symptoms elicited by microbial endotoxins [35] prior to the use of antibiotics.

Thus, the inhibitors of 17beta-HSD5 are highly interesting potential therapeutic agents that guarantee the efficacy of the GC to prevent the host worsening and dying [36] before the clearance of the bacteria by the antibiotics. Therefore, in this study we investigated if the 17 beta-HSD5: 4XVE will be susceptible to inhibition by compound (**6a**).

**Figure 7:**
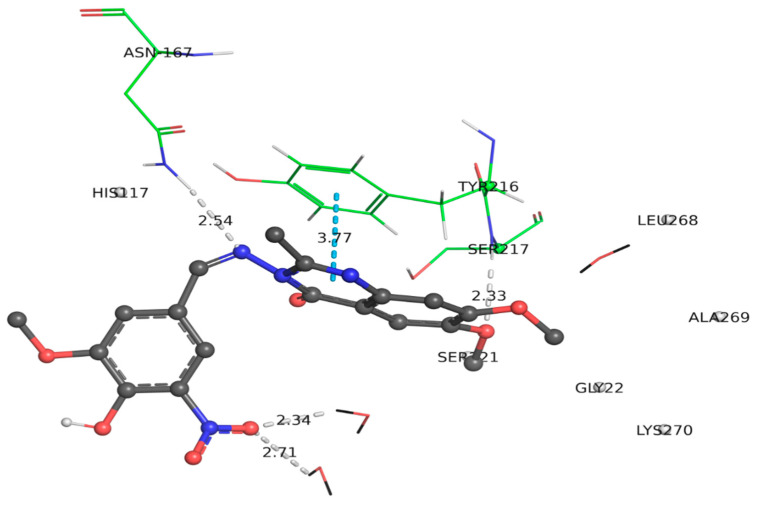
Docking pose of molecule (**6a**) in the active site of 17 beta-HSD5 (pdb: 4XV).

As depicted in Figure 7, substrate (**6a**) has adopted a hook shape inside the active site of the 17beta-HSD5 enzyme. The quinazolinone ring is responsible for the pi−pi interactions with the Tyr216 residue (distance: 3.77 Å); thus, its retention can be beneficial in future modifications. Moreover, the nitrogen atom attached to the quinazolinone ring is very important for fitting of the chemical substrate inside the enzymatic cavity through H-bonding with Asn167 (distance 2.54 Å). The Ser217 residue and the -OMe group on the quinazolinone core are connected through the H-bond with a distance of 2.33 Å. Moreover, the nitro group on the aryl side chain is accountable for the H-bonding interactions with two water molecules present inside the active site of the target enzyme.

#### 2.3.4. Docking of Compound (**6d**) within the Active Site of COX-2 

**Figure 8:**
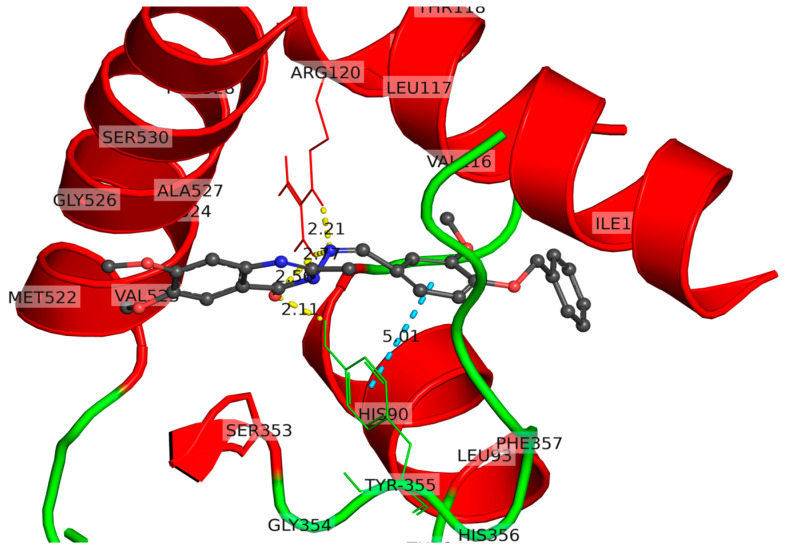
Three-dimensional docking pose of molecule (**6d**) in the active site of COX-2.

Docking of (**6d**) within the active site of COX-2 (pdb: 3NT1) was performed to identify the binding interactions between them. The docking poses shown in Figure 8, Figures S2 and S3 indicated that the ligand (**6d**) possessed H-bond interactions with Tyr355 and Arg120 via the oxygen and nitrogen atoms of its amino quinazolinone core. Moreover, there is pi−pi interaction involving the 4-(benzyloxy)-3-methoxyphenyl moiety and Tyr355. Therefore, the quinazolinone and 4-(benzyloxy)-3-methoxyphenyl rings are important for establishing interactions with the receptor. Further analysis demonstrated that the azomethine moiety −N=CH−, which acts as a useful linker between the quinazolinone core and the aromatic side group, is involved in H-bonding with Arg120. Interestingly, the molecule adopts a linear shape inside the cavity, although the quinazolinone ring and the vicinal aromatic ring are perpendicular to each other. Thus, this −N=CH− linker enhanced the flexibility of the molecule. One of the −OMe groups on the quinazolinone core is responsible for the hydrophobic interactions with the Met522 residue. Additionally, the deep insertion of the molecule in the receptor-binding site is responsible for the establishment of a number of polar and van der Waals interactions.

### 2.4. The Five Metrics of Lipinski’s Rule and ADMET Profiling of the Synthesized Compounds

The drug-likeness characteristics (Lipinski’s rule of five) and the expected in vivo pharmacokinetic and toxicity properties of the synthesized compounds were predicted via in silico ADMET evaluation using the pK-CSM: graph based-signatures platform [37] (http://biosig.unimelb.edu.au/pkcsm/prediction). These assessments are very useful at preclinical stage of drug discovery to identify the best candidates for further lead optimization, and to exclude those with undesirable physicochemical and biological properties.

The Lipinski rule, states that “a compound is likely to be impermeable or badly absorbed if it violates more than 2 of the following criteria: a molecular weight < 500 Da, cLog*P* (calculated Log lipophilicity*)* ≤ 5, topological polar surface area (TPSA) < 150 Å^2^, number of H-bond acceptors (expressed as the sum of Ns and OS) ≤ 5, and number of H-bond donors (expressed as the sum of OHs and NHs) ≤ 5 [38]. 

Further, it was reported that a compound’s bioavailability will be very high if TPSA value ≤ 140 Å^2^ and rotatable bonds ≤ 10 [39].

As shown in Table S5 in the Supplementary Materials, all the synthesized compounds were found to adhere to the molecular weight and cLogP metrics. Contrarily, compounds (**4c**, **6a**, **6b**, **6d,** and **6e**) failed to comply with the parameter of number of H-bond acceptor groups and compounds (**6a**, **6b**, **6c**, **6d**, and **6e**) did not conform to permissible TPSA values. Despite that the compounds (**4c**, **6a**–**e**) violated the rule, all the synthesized compounds would be expected to be orally bioavailable as none of them had violated more than two criteria [38].

The results of the ADMET predictions of all the synthesized compounds are presented in Tables S6−S10 in the Supplementary Materials.

For simplification, only the results of the most biologically active candidates will be discussed in details.

#### 2.4.1. Absorption

Since oral administration is the preferred route for drug delivery, thus the first phase in the journey of a drug molecule through the body to reach its target site is absorption. The fundamental properties influencing the in vivo absorption of a drug candidate, including the water solubility, permeability across the gastrointestinal tract lining, and binding to P-glycoprotein (P-gp) efflux proteins or inhibition of P-gpI or II, were predicted and are presented in Table S6.

In principle the overall water solubility of a drug is a composite sum of the contributions of all the functional groups within the molecule. These molecular entities may confer water solubility via H-bond formation with water molecules (such as −NH_2_, −OH, and −OCH_3_) and/or ionization of N^+^H_4_ and COOH groups.

Conversely, other groups could confer lipid solubility, such as aromatic groups and halogens (Cl, Br, and I; the effect of F can vary) [40]. Thus, according to the predicted values of aqueous solubility(expressed as log units of molar solubility, log mol/L), it is important to note that the starting materials (**1**, **2**, and **5a**) show the highest log mol/L values (−2.287, −2.486, and −2.683, respectively), compared to the Schiff’s bases, which implies that introduction of the aryl groups induces an increase in the lipophilic character of the molecules.

All the biologically active Schiff’s bases, show moderate-to-high solubility with log units spanning −5.419 (**6e**) to −3.127 (**4c**). The quinoxalinone derivatives are likely to be more soluble than the quinazolinone analogs because they have an additional −NH group capable of H-bonding. Thus, the quinoxalinone and quinazolinone derivatives (**4c**, **4d**) and (**6e**, **6d**) show the highest and the lowest log units, respectively. The reduced water solubility of the latter derivatives may be attributed to the bulkiness and the flexibility of the benzyloxy group, which may retard the capability of H-bond formation with water molecules.

Conversely, the presence of more groups capable of H-bond formation (−OH, −OCH_3_, and −NO_2_) in the aryl group in compound (**4c**), enhances its solubility relative to (**4d**), which has only the −COOH group. Similarly, derivatives (**6a**) and (**6b**) are the most soluble among quinazolinone candidates. Despite their structural similarities, (**6f**) is slightly less soluble than (**6g**), which may be attributed to the replacement of Cl atom by F, which has higher affinity for hydrogen bond formation with a nearby water molecule.

As the intestine is the primary site for absorption of an orally administrated drug, the proportion of compounds that are absorbed through the human small intestine are predicted by assessing two parameters: the Caco2 permeability (expressed as the logarithm of the apparent permeability coefficient, log Papp in cm/s) and the human intestinal absorption (HIA in %).

The Caco2 permeability predicts the rate of flux of a compound across Caco-2 cell monolayers (the human epithelial colorectal adenocarcinoma cells), which is commonly used as an in vitro model of the human intestinal mucosa.

The predicted data show that the quinazolinone derivatives would exhibit better Caco2 permeability, than the quinoxalinones. This prediction is in agreement with the increased tendency for H-bond formation with surrounding water molecules by quinoxalinone derivatives, as discussed above, which negatively impacts on the degree of permeability.

Therefore, compounds (**4d**) and (**6g**) are predicted to show the lowest and highest log Papp values of −0.245 and 1.388 in 10^−6^ cm/s, respectively. As regarding to compound (**6d**), it shows high Caco2 permeability with log Papp rate of 1.314 in 10^−6^ cm/s, which is well above the recommended value of 0.90 [37].

Moreover, the same set of the bioactive compounds are expected to show moderate-to-high HIA ability ranging from 59.924%, which is the lowest value among all the synthesized compounds and it is exhibited by (**4d**) to 99.584% that is displayed by (**6d**), which is better than the recommend value of 30% [37]. The low permeability and %HIA values, which are predicted for compound (**4d**), (among all the synthesized compounds), can be attributed to the existence of strong H-bonds between its highly polar −COOH group and the water molecules [41].

Similarly, among quinazolinone derivatives; (**6a**) and (**6b**) are predicted to exhibit the lowest Caco2 permeability and %HIA due to their strong H-bond formation capabilities.

From these results, the negative impact of the strong H-bonding capacity on both permeability and % HIA can be interpreted in terms of the fact that for a compound to be passively absorbed and able to penetrate a biological membrane, its H-bonds with the water molecules in the aqueous environment have to be broken to allow the compound to adopt the optimal angle and intermolecular distance to achieve maximal binding potency with biological receptors. However, as the strength of H-bond increases, the membrane penetration decreases, presumably due to a high-water de-solvation penalty required for moving from the aqueous surrounding to the lipophilic membrane interior.

Another important parameter that significantly influences the absorption of a chemical in the gastrointestinal tract is whether or not it will be a P-glycoprotein (P-gp) substrate or an inhibitor as recommend by the US food and drug administration (FDA). 

P-gp is a transporter protein that found normally in many body organs and tissues, including the stomach, placenta kidney, brain, liver, and others. Generally, P-gp acts as a transporter to efflux the chemicals from the cells, thus preventing their intracellular accumulation to protect the cells against xenobiotics and toxins. In gastrointestinal tract cells, P-gp blocks the absorption of their substrates after oral administration, and in the brain, it prevents the entry of antiviral drugs [42].

However, the efflux ability may result in chemotherapeutic resistance and reduce the effectiveness of certain drugs such as anticancer, antibiotic, HIV protease inhibitors, and others [43]. In various human tumors this protein is commonly overexpressed and acts to lower the drug’s concentration at the intracellular target site to subtherapeutic level resulting in resisting apoptosis and failure of the treatment by many anticancer drugs such as vinblastine and daunorubicin [44].

Conversely, the combination of an inhibitor for P-gp with the chemotherapeutic drug is proposed as a promising approach that would have on patient outcomes concerning relapse free and overall survival [45].

Another serious aspect of a compound’s P-gp inhibitory activity is its ability to induce drug–drug interactions (DDIs), which are commonly associated with serious side effects that may lead to mortality. DDIs occur when a P-gp inhibitor is coadministrated with a P-gp substrate, resulting in the accumulation of the substrate drug, which lead to occurrence of drug-induced toxicity. The obtained results, reveal that the anti-CRC candidate (**6d**) is not a potential P-gp substrate, this suggests that the transportation of this compound is not dependent on P-gp pathway. Contrarily, (**6d**) is likely to be an inhibitor for both of P-gpI and P-gpII; thus, it may be considered as a chemotherapeutic candidate that can prevent or reverse the multidrug resistance [42], although DDIs have to be considered with coadministered substrates.

Similarly, among the antibacterial candidates (**2**, **4a**, **4b**, **4c**, **4d**, **5b**, **6a**, **6b**, **6d,** and **6e**), compounds (**6e** and **6d**) do not have any affinity to P-gp. However, they are potential inhibitors; thus, they may be considered as promising leads for the discovery of new antibacterial agents.

Meanwhile compound (**6b**) is neither a potential ligand of P-gp nor an inhibitor of P-gpI and P-gpII. Therefore, there is no possibility for DDIs between (**6b**) and P-glycoprotein substrates or inhibitors.

Notably, to indicate that although compounds (**6d** and **6e**; **6a**) showed potent in vitro anti-COX-2 inhibitions, derivatives (**6e** and **6a**) failed to produce significant cytotoxic effects against the tested cell lines, which may be attributed to their lowered Caco-2 permeability and cellular absorption properties compared to those of compound (**6d**) as demonstrated by our in silico prediction results (Table S6). In addition, the active cytotoxic candidate is not a likely substrate for P-gp transporter, whereas (**6a**) is, therefore the latter compound may be pumped out of the cells, which negatively impacts its cytotoxic effect.

Although oral administration is the more preferred route, some drugs are dermally administrated. The skin permeability is predicted in terms of log permeability coefficient (log Kp) and a compound with a log Kp of > −2.5 has a low skin permeability. The studied compounds are predicted to have log Kp values ranging from −2.746 (**6a**) to −2.433 (**6g**).

#### 2.4.2. Distribution

After oral absorption, the drug enters the systematic circulation to be distributed throughout the body. Three main measures have been predicted to estimate tissue distribution: the volume of distribution at steady-state (Vdss), fraction unbound in plasma (*f*_u,p_), and uptake by blood brain barrier (BBB) and central nervous system (CNS), as shown in Table S7.

The Vdss is an important descriptor of the drug concentration in plasma compared to the total drug concentration in the body and it provides an indication about the capability of the drug to penetrate the tissues and body organs. The Vdss is given as the log L/kg, and it is considered low if log VDss < −0.15 and high if log VDss > 0.45 [37]. Among the synthesized compounds, the smallest predicted log Vdss values are −1.368 and −1.153, which are demonstrated by compounds (**4d**) and (**6b**), respectively, implying that they are confined to the plasma and they are not distributed to the body orangs and tissues. Conversely, (**6g**) is expected to exhibit the highest log Vdss value among the bioactive compounds (−0.088), which indicates that it has a broad distribution.

This value may suggest that (**6g**) has optimal lipophilic characters than (**4d**); thus, it has a high affinity toward lipids, proteins, and cellular membranes. The lipophilicity of quinazolinone (**6g**) may be attributed to the presence of the non-polar aryl group (5-ethyl thienyl moiety) compared to quinoxalinone (**4d**), which has the 4-benzoic acid moiety. Additionally, the presence of F atom in the (**6g**) contributes positively to VDss [46].

Among COX-2 inhibitors, (**6d**) has a relatively higher Vdss value of −0.387 than (**6a**, −0.433), which is lower than that of (**6e**, −0.173).

The (*f*_u,p_) has great impact on drug efficacy. This is because, only the unbound drug is capable of binding with its molecular target (receptor), thus this parameter determines the drug concentration at the active sites [47]. Among the investigated active compounds; (**6d**) and (**4d**) have the lowest and the highest values of 0.103 and 0.163 fraction unit, respectively.

The knowledge of the blood brain barrier (BBB) and CNS penetration abilities are of significant importance in drug discovery, particularly for peripherally acting drugs, whose exposure to the brain should be limited to minimize the potential psychotropic risks.

The extent of drug penetration through the BBB is essentially quantified by two standard experimental measures. The most common used descriptor is the logarithm of the ratio of steady-state concentration of the drug in the brain to that in the blood (log BB); thus, this parameter predicts the total brain exposure to drugs, at a steady state. Based on this measure, if a compound has a logBB > 0.3, it will penetrate BBB, however, it poorly will be distributed to the brain if it has a logBB < −1.

The second measure is the logarithm of the permeability surface-area product (log PS), which describes the uptake clearance across the BBB into the brain [48]. For a given compound, it will be able to penetrate the CNS if it has a logPS value > −2, while a compound with a logPS < −3, it is considered unable to penetrate CNS.

According to these measures, the studied compounds are expected to display low-to-moderate abilities to penetrate the BBB (ranging from log BB = −1.399 by **6a** to 0.331 by **6g**) and/CNS (ranging from log PS = −3.304 by **6a** to −1.394 by **6g**) in the order of (**6a** < **6d** < **4c** < **6b** < **4d** < **6e** < **6g**)/and (**6a** < **6b** < **6d** < **4c** < **4d** < **6e** < **6g**), respectively. Therefore, compound (**6d**), which is predicted to have log BB = −1.1 and log PS = −3.046, would be relatively safe, compared to compounds (**6e** and **6g**), which would induce CNS side effects.

The high penetration capability of (**6g**) to the brain and CNS may be attributed to its increased lipophilicity, accompanied by reduced overall hydrogen bonding propensity compared to the rest of the compounds.

#### 2.4.3. Metabolism

Once a compound penetrates the gastrointestinal tract it passes through the portal vein to the liver for phase I hepatic metabolism via oxidations, reductions, and dealkylation reactions. The main group of hepatic enzymes that is responsible for these transformations are the cytochrome P450 (CYP450) family of enzymes. Five major cytochrome P450 (CYP) isoforms: CYP1A2, CYP2C19, CYP2C9, CYP2D6, and CYP3A4 are mainly considered responsible for most of the metabolic reactions that enable the body to eliminate a drug. Therefore, it is very crucial for the drug molecule to resist these enzymes (i.e., to show metabolic stability) [49].

The predicted in silico data for the expected metabolic interactions of the synthesized compounds (Table S8) indicate that only (**4d**) is a possible substrate for CYP2D6, while compounds (**4c**), (**6a**), (**6c**), (**6d**), and (**6e**) are expected to be metabolized by CYP3A4, however, (**6b**) will not be metabolized by any of these isoforms. In drug discovery, it is advantageous for a new chemical entity to be a CYP3A4 rather than a CYP2D6 substrate, because the later enzyme is encoded by genes that exhibit polymorphism among individuals, which significantly impact the drug-metabolizing enzymatic function. Importantly, the alteration of the CYP2D6 function has been associated with both adverse drug reactions and reduced therapeutic efficacy at standard doses [50].

In particular, the clinical consequences of CYP2D6 polymorphism on the metabolism of anticancer drugs have been increasingly studied. For example, based on a prospective multicenter study, it was predicted that the response to tamoxifen in women with breast cancer depends on the *CYP2D6* genotype [51].

Conversely, the genetic diversity in the genes encoding CYP3A4 proteins most of them are very rare and unlikely to impact its enzymatic activity and the pharmacokinetic parameters of drug substrates [52,53].

Furthermore, it is predicted that the same set of bioactive candidates will display varied inhibitory effects on the different isoforms (Table S8). Consequently, there is a possibility for the DDIs of the possible inhibitors to occur if they are coadministered with drug substrates leading to the alternation of the pharmacokinetic properties of the substrates. Furthermore, the suppression of these isoenzymes may result in toxic effects caused by the accumulation of a drug substrate or its metabolite in non-target tissue [54].

Concerning, (**6d**) it is predicted to be an inhibitor for CYP2C19, CYP2C9, and CYP3A4. Therefore, this antiCRC candidate may block the metabolism of proton pump inhibitors, and antimalarial, antifungal, antioestrogenanesthetic, and analgesic drugs via the inhibition of CYP2C19. It may also halt the metabolism of anticlotting agents, antiseizure, medications of type-II diabetes, and antihypertensive, and NSAIDs via the suppression of CYP2D9. Additionally, it is expected to halt the oxidation of steroids, fatty acids, and antibiotics, and hormone synthesis and breakdown through CYP3A4 inhibition.

Conversely, candidate (**6d**) will not halt the liver metabolism or biotransformation of antihypersensitive drugs, β-blockers, antihistamines, antidepressant, and antiemetic drugs [55] as it is not expected to inhibit CYP1A2 or CYP2D6.

Whereas derivatives (**4c**, **4d**, **6e,** and **6g**) are considered as perpetrators and expected to have in vivo DDIs with the drugs undergoing CYP1A2-mediated metabolism such as cardiovascular drugs (propranolol and verapamil), antipsychotics (clozapine and olanzapine), antidepressants (duloxetine, agomelatine, and mirtazapine), NSAIDs (phenacetin), phosphodiesterase inhibitors (theophylline), and CNS stimulants (caffeine) [56].

#### 2.4.4. Excretion

The prediction of the removal rate of each drug candidate from the systemic circulation by all methods (renal clearance; hepatic clearance via metabolic loss; and loss into breast milk, sweat and saliva) is achieved by determining of the expected total clearance parameter (CLs), which is expressed as log mL/min/kg.

The CLs is inversely proportional to a compound’s in vivo half-life time, thus a higher CLs value indicates that a compound is more rapidly removed from the body by any method, which may impact its potency (how tightly a compound binds to its target). Thus CLs, together with the volume of distribution, are responsible for the determination of the dosing frequency of a drug [57].

The CL data presented in Table S9, show that molecules (**6g**) and (**6e**) possess relatively low total CL values, whereas, compounds (**6a**), (**6b**), and (**6d**) have high values.

Additionally, the prediction results of a compound’s tendency to be substrates for renal transporter; organic cation transporter 2 (OCT2) [58] indicate that all the studied compounds except for (**6g**), are not possible ligands, as shown in Table S9. This finding suggests that the cytotoxic candidate (**6d**) would not be accumulated in the kidney and would not induce DDIs [59] in the renal proximal tubular cells. Accordingly, (**6d**) is not a potential risk factor for kidney damage and nephrotoxicity.

#### 2.4.5. Toxicity Profile

Several toxicity parameters are predicted to assess the potential safety liabilities associated with the synthesized compounds (Table S10). Therefore, the compounds were screened for their inhibitory activity on two of the voltage-gated potassium channels encoded by the human ether a-go-go-related gene (hERG), which are the hERGI (encoded by KCNH2 gene) found mainly in the heart, and the hERGII (now is referred to as Kv11.2 and encoded by KCNH6 gene) found in β cells of the pancreas. The screening results against hERGI show that none of the compounds is a potential inhibitor (i.e., no risk for long QT syndrome, which leads to ventricular arrhythmia and sudden cardiac arrest) [60]. In contrast, among the bioactive derivatives, (**6a**), (**6d**), and (**6e**) are likely to inhibit the hERGII voltage gate channel, which may lead to glucose metabolic disturbances via β-cells dysfunction [61].

Other parameters, including the overall level of mutagenicity (predicted by Ames toxicity test), hepatotoxicity and skin sensitization by the compounds are qualitatively evaluated using the categorical classification: yes or no. The predicted results for the hepatotoxicity indicate that only compounds (**4d**) and (**6d**) are nontoxic, whereas, compounds; (**4c**), (**6a**), (**6b**), (**6e**), and (**6g**) are toxic. Moreover, all the studied compounds are not mutagenic chemicals or skin sensitizers.

Further assessment and ranking (numerical) of the toxicity risks of the compounds are conducted on *Tetrahymena pyriformis* protozoan, and the flathead minnow fish model. The concentrations of the chemicals (log µg/L) at which the proliferation of *T. pyriformis* is inhibited are predicted and thus the compounds can be ranked as follows: (**6b = 6d**) < (**4d**) < (**6e**) < (**6a**) < (**4c)** < (**6g**). Therefore, compounds (**6b**) and (**6d**) would be the least toxic.

Similarly, the log median lethal concentration values (log LC_50_ expressed as log mM) of the compounds against flathead minnows are estimated. Accordingly, the compounds’ toxicity order is expected to be as follows: (**6e**) < (**6d**) < (**6g**) < (**6a**) < (**6b**) < (**4d**) < (**4c**). 

Lastly, the maximum tolerated dose (in human) expressed in mg/kg/day, which provides information about the compound’s concentration above which its efficacy is not improved, or its adverse effects outweigh the benefits are predicted. For compound (**6d**) the maximum tolerated dose is calculated to be 0.564 mg/kg/day, which is relatively high compared to the lowest value of −0.232 (**2**) and the highest value of 0.819 (**4d**). 

In addition, the potential risks posed to the human health after acute and chronic exposure to the synthesized compounds are evaluated by the prediction of the median lethal dose (LD_50_, mol/kg) and the lowest-observed-adverse-effect level (LOAEL, log mg/kg-bw/day) in rat models. It is found that compound (**4d**) possessed the highest LD_50_ (3.203) and LOAEL (2.901) values among all the studied compounds. For compound (**6d**), the LD_50_ was found to be 2.711 mol/kg and LOAEL is 1.077 (log mg/kg-bw/day).

## 3. Experimental

### 3.1. Chemistry

#### 3.1.1. Instrumentation

All melting points were measured on the Gallenkamp melting point apparatus and are uncorrected. The IR spectra were recorded in wave number (ν, cm^−1^) on a Perkin Elmer FT spectrophotometer (Spectrum BX 1000) using potassium bromide (KBr) disks. The NMR experiments (^1^H- and ^13^C-NMR) were carried out on a JEOL ECP NMR spectrometer operating at 300 MHz or 500 MHz; the chemical shifts were expressed in (ppm) downfield from tetramethylsilane (TMS, the internal standard); the coupling constants (*J*) were expressed in Hz, using deuterated dimethyl sulfoxide (DMSO-*d_6_*) or chloroform as the solvent; the splitting patterns were designated as s (singlet), br. s (broad singlet), d (doublet), dd (doublet of doublets), t (triplet), q (quartet), and m (multiplet). The mass spectra were recorded on a direct probe controller inlet part to single quadrupole mass analyzer in (Thermo Scientific GCMS) model ISO Lt using the Thermo X-Calibur software at the regional center for Mycology and Biotechnology at Al-Azhar University, Nasr City, Cairo, Egypt. The C, H, N, and S contents of the new compounds were also determined in the regional center for Mycology and Biotechnology at Al-Azhar University. The biological evaluations of the products were carried out at King Saud University, Riyadh, Saudi Arabia.

#### 3.1.2. General Procedures for the Synthesis of Schiff’ Bases (**4a**–**e**) and (**6a**–**g**) 

The precursors (**1**), (**2**), and (**5a**-**c**) were reported previously [62,63,64,65,66].

An equimolar mixture (0.002 mol) of 3-hydrazineylidene-3,4-dihydro quinoxalin-2(1*H*)-one (**2**) or 3-amino-2-methyl-3*H*-quinazolin-4-one derivatives (**5a**–**c**), and the appropriate aromatic aldehyde (**3a**–**g**): 5-chloro vanillin, 5-idodo vanillin, 5-nitro vanillin, 4-formylbenzoic acid, 6-methoxy-2-naphthaldehyde, 4-(benzyloxy)-3-methoxybenzaldehyde, or 5-ethylthiophene-2-carbaldehyde, in absolute ethanol (20 mL) containing catalytic amount of acetic acid (3 mL), was refluxed sequentially for 10 h. The separated solid in each case was collected by filtration, washed with water, air-dried, and recrystallized from the appropriate solvent to afford the corresponding Schiff’s base derivatives (**4a**–**e**) and (**6a**–**g**).

***3-[N’-(3-Chloro-4-hydroxy-5-methoxy-benzylidene)-hydrazino]-1H-quinoxalin-2-one*** (**4a**)

Orange powder (EtOH/CHCl_3_), yield (59%), m.p. 281–282 °C, ν_max_ (KBr)/cm^−1^ 3500 (OH), 3320 (2 × NH), 3099 (CH-aromatic), 2954 (CH-aliphatic), 2847, 1677 (C=O), 1616 and 1581 (2 × C=N), 1580, 1498 and 1417 (C=C), 1367, 1285, 1214, 1149, 1101, 1048, 998, 926, 898, 852, 748, 679, 619, 589, 528, 464; ^1^H-NMR (300 MHz, DMSO-*d_6_*) *δ*_H_: 12.42 (1H, br. s, NHCO), 11.19 (1H, br. s, NH), 9.95 (1H, s, OH), 8.46 (1H, s, CH=N), 7.52 (1H, s, CH-aromatic), 7.36–7.06 (5H, m, 5 × CH-aromatic), 3.91 (3H, s, OCH_3_); ^13^C-NMR (150 MHz, DMSO-*d_6_*) *δ*_C_: 156.95 (C=O), 155.33, 151.26 (2 × C_q_-O), 149.32, 146.45 (C_q_=N and HC=N), 146.33, 145.57, 144.77, 133.24, 129.05, 126.83, 125.87, 124.99, 123.91,121.22, 120.51, 115.44, 110.67, 108.50 (6 × CH-aromatic and 4 × C_q_-aromatic), 57.70 (OCH_3_); MS (EI) *m/z* (%) [M^+^ + H] 345.25 (12.07), [M^+^] 344.11 (100), 331.07 (19.35), 320.99 (12.42), 310.14 (16.72), 300.12 (60.91), 293.48 (5.89), 287.44 (36.10), 286.21 (49.86), 269.45 (29.73), 251.87 (10.11), 241.07 (13.09), 220.30 (9.81), 209.90 (24.46), 200.36 (22.71), 184.03 (17.49), 182.91 (34.84), 173.98 (13.42), 170.95 (11.84), 162.12 (67.28),155.01 (17.23), 143.10 (27.13), 133.14 (60.26), 126.33 (25.77), 125.02 (36.93), 115.97 (12.39), 108.69 (11.87), 96.37 (22.71), 89.13 (21.75), 59.97 (21.43), 42.23 (31.10). Anal. Calcd. for C_16_H_13_ClN_4_O_3_: C, 55.74, H, 3.80, N, 16.25. Found: C, 55.57, H, 3.69, N, 16.15.

***3-[N’-(4-Hydroxy-3-iodo-5-methoxy-benzylidene)-hydrazino]-1H-quinoxalin-2-one*** (**4b**)

Orange powder (EtOH/CHCl_3_), yield (84%), m.p. 254–255 °C, ν_max_ (KBr)/cm^−1^ 3488 (OH), 3318 (2 × NH), 3009 (CH-aromatic), 2850 (CH-aliphatic), 1672 (C=O), 1618 and 1580, (2 × C=N), 1492 and 1412 (C=C), 1364, 1272, 1214, 1183, 1150, 1095, 1040, 997, 931, 863, 804, 747, 676, 589, 528, 464; ^1^H-NMR (300 MHz, DMSO-*d_6_*) *δ*_H_: 12.36 (1H, br. s, NHCO), 11.11 (1H, br. s, NH), 10.08 (1H, br. s, OH), 8.45 (1H, s, CH=N) 7.71–7.10 (6H, m, 6 × CH-aromatic), 3.90 (3H, s, OCH_3_); ^13^C-NMR (150 MHz, DMSO-*d_6_*) *δ*_C_: 151.27 (C=O), 148.34, 147.64, 146.40, 146.16 (HC=N, C_q_=N, 2 × C_q_-O), 133.25, 129.88, 129.02, 128.48, 125.85, 124.96, 123.90, 115.42, 109.81, 85.03 (6 × CH-aromatic and 4 × C_q_-aromatic), 56.61 (OCH_3_); MS (EI) *m/z* (%) [M^+^ + H] 437.12 (2.34), [M^+^] 436.09 (11.81), [M^+^ − H] 435.23 (3.62), 310.18 (11.72), 294.09 (2.09), 277.01 (2.63), 266.15 (2.40), 253.89 (8.84), 249.97 (1.23), 187.03 (3.92), 175.04 (2.16), 161.15 (78.06), 150.17 (5.36), 141.94 (7.90), 133.17 (74.89), 127.00 (47.06), 118.14 (33.52), 105.17 (60.45), 90.16 (84.44), 79.16 (100.00), 78.15 (75.99), 63.16 (76.97), 51.15 (68.46), 43.12 (30.45). Anal. Calcd. for C_16_H_13_IN_4_O_3_: C, 44.06, H, 3.00, N, 12.84. Found: C, 44.34, H, 3.17, N, 13.08.

***3-[N’-(4-Hydroxy-3-methoxy-5-nitro-benzylidene)-hydrazino]-1H-quinoxalin-2-one*** (**4c**)

Dark red powder (EtOH/CHCl_3_), yield (81%), m.p. 261–262 °C, ν_max_ (KBr)/cm^−1^ 3405 (OH), 3304 (2 × NH), 3104 and 3067 (CH-aromatic), 2947 and 2890 (CH-aliphatic), 2845, 2341, 2372, 1671 (C=O), 1614 and 1575 (2 × C=N), 1535, 1500 and 1466 (C=C), 1404, 1358 (NO_2_), 1317, 1277, 1216, 1143, 1084, 1057, 1004, 919, 878, 854, 788, 756, 716, 680, 648, 616, 584, 465; ^1^H-NMR (300 MHz, DMSO-*d_6_*) *δ*_H_: 12.24 (1H, s, NHCO), 11.20 (1H, br. s, NH), 8.53 (1H, s, CH=N), 7.81-7.69 (2H, m, 2 × CH-aromatic), 7.53 (1H, s, CH-aromatic), 7.30–7.00 (3H, m, 3 × CH-aromatic), 3.97 (3H, s, OCH_3_); ^13^C-NMR (150 MHz, DMSO-*d_6_*) *δ*_C_: 150.16 (C=O), 146.25, 144.40 (HC=N, C_q_=N, 2 × C_q_-O), 137.58, 125.85, 124.50, 123.77, 116.25, 115.42, 113.31, 113.06 (6 × CH-aromatic and 4 × C_q_-aromatic), 57.12 (OCH_3_); MS (EI) *m/z* (%) [M^+^ + H_2_] 357.08 (3.10), [M^+^ + H] 356.02 (3.99), [M^+^] 355.21 (18.87), [M^+^ − H] 354.34 (2.10), 337.45 (3.76), 307.22 (6.15), 293.15 (3.67), 240.14 (2.23), 220.06 (11.40), 186.65 (11.42), 174.24 (3.49), 161.09 (58.74), 146.08 (4.44), 133.15 (35.87), 118.19 (33.17), 106.17 (21.09), 91.13 (57.27), 76.14 (37.36), 51.17 (100.00), 45.06 (36.85), 43.18 (22.18). Anal. Calcd. for C_16_H_13_N_5_O_5_: C, 54.09, H, 3.69, N, 19.71. Found: C, 54.31, H, 3.85, N, 19.93.

***4-[(3-Oxo-3,4-dihydro-quinoxalin-2-yl)-hydrazonomethyl]-benzoic acid*** (**4d**)

Green powder (EtOH/CHCl_3_), yield (83%), m.p. 379–380 °C, ν_max_ (KBr)/cm^−1^ 3484 (OH), 3315 (2 × NH), 3108 and 3011 (CH-aromatic), 2962, 2897, 2853, 2606, 2466, 2374, 1676 (2 × C=O), 1617 and 1577 (2 × C=N), 1503, 1475 and 1416 (C=C), 1389, 1354, 1314, 1235, 1178, 1086, 1017, 937, 866, 772, 745, 695, 647, 596, 548, 507, 471, 391; ^1^H-NMR (300 MHz, DMSO-*d_6_*) *δ*_H_: 12.44 (1H, br. s, NHCO), 11.42 (1H, br. s, NH), 8.64 (1H, s, CH=N), 8.40–7.80 (4H, m, 4 × CH-Ar), 7.56 (1H, d, *J* = 5.1 Hz, CH-quinoxalinone), 7.33–7.01 (3H, m, 3 × CH-quinoxalinone); ^13^C-NMR (150 MHz, DMSO-*d_6_*) *δ*_C_: 167.42 (carboxylic C=O), 151.23 (lactamic C=O), 146.54, 145.78 (HC=N, C_q_=N), 139.26, 133.10, 131.60, 130.25, 129.26, 127.25, 126.09, 125.41, 124.00, 115.50 (8 × CH-aromatic and 4 × C_q_-aromatic); MS (EI) *m/z* (%) [M^+^] 308.95 (16.85), 288.60 (17.20), 262.31 (44.76), 254.12 (16.07), 248.08 (40.46), 236.19 (32.17), 218.01 (8.17), 210.27 (21.28), 203.18 (100.00), 174.89 (24.58), 161.91 (35.21), 152.99 (44.45), 137.35 (11.88), 117.73 (28.21), 97.25 (29.64), 89.03 (44.19), 68.50 (33.31), 47.36 (58.11), 40. 43 (52.13). Anal. Calcd. for C_16_H_12_N_4_O_3_:C, 62.33, H, 3.92, N, 18.17, O, 15.57. Found: C, 62.51, H, 4.51, N, 18.33.

***3-[N’-(6-Methoxy-naphthalen-2-ylmethylene)-hydrazino]-1H-quinoxalin-2-one*** (**4e**)

Orange powder (EtOH/CHCl_3_), yield (80%), m.p. 286–287 °C, ν_max_ (KBr)/cm^−1^ 3304 (2 × NH) 3109 and 3056 (CH-aromatic), 2978 and 2938 (CH-aliphatic), 2818, 2671, 1675 (C=O), 1620 and 1574 (2 × C=N), 1529 and 1500 (C=C), 1385, 1332, 1266, 1243, 1208, 1175, 1119, 1088, 1026, 926, 977, 806, 752, 651, 623, 597, 545, 475; ^1^H-NMR (300 MHz, DMSO-*d_6_*) *δ*_H_: 12.47 (1H, br. s, NHCO), 11.33 (1H, br. s, NH), 8.73 (1H, s, CH=N), 8.01–7.82 (4H, m, 4 × CH-aromatic), 7.58 (1H, d, *J* = 3.9 Hz, CH-naphthalene), 7.35 (1H, s, CH-naphthalene), 7.29–7.15 (4H, m, 4 × CH-aromatic), 3.88 (3H, s, OCH_3_); ^13^C-NMR (75 MHz, DMSO-*d_6_*) *δ*_C_: 158.44 (C=O), 151.24 (C_q_-O), 147.38, 146.48 (C_q_=N and HC=N) 135.40, 133.30, 130.63, 130.19, 129.05, 128.58, 128.35, 127.64, 125.78, 124.92, 123.85, 123.74, 119.39, 115.39, 106.66 (10 × CH-aromatic and 5 × C_q_-aromatic), 55.60 (OCH_3_), MS (EI) *m/z* (%) [M^+^ + H] 345.12 (2.97), [M^+^] 344.23 (25.39), 316.17 (8.74), 301.19 (6.72), 300.21 (14.45), 186.17 (2.25), 184.13 (10.65), 183.15 (18.63), 161.14 (100.00), 160.20 (6.52), 157.20 (10.83), 142.18 (16.40), 134.13 (39.99), 133.17 (75.14), 132.27 (15.98), 127.20 (26.25), 126.23 (15.46), 119.08 (7.94), 118.14 (26.31), 106.19 (28.87), 105.20 (61.86), 91.16 (33.64), 90.15 (83.76), 77.14 (56.99), 76.21 (24.77), 51.16 (28.85), 45.18 (27.68), 43.16 (16.82). Anal. Calcd. for C_20_H_16_N_4_O_2_: C, 69.76, H, 4.68, N, 16.27. Found: C, 69.57, H, 4.79, N, 16.44.

***3-[(4-Hydroxy-3-methoxy-5-nitro-benzylidene)-amino]-6,7-dimethoxy-2-methyl-3H-quinazolin-4-one*** (**6a**)

Yellow powder (MeOH/CHCl_3_), yield (62%), m.p. 260–261 °C, ν_max_ (KBr)/cm^−1^ 3268 (OH), 3089 and 3008 (CH-aromatic), 2940 and 2840 (CH-aliphatic), 2049, 1665 (C=O), 1608 (C=N), 1549 (C=N), 1501 and 1465 (C=C), 1394, 1336 (NO_2_), 1309, 1270, 1247, 1212, 1164, 1108, 1051, 1025, 980, 918, 877, 837, 810, 776, 733, 703, 623, 601, 550, 439; ^1^H-NMR (300 MH_Z_, DMSO-*d_6_*) *δ*_H_: 8.89 (1H, s, CH=N), 8.02 (1H, s, CH-aromatic), 7.81 (1H, s, CH-aromatic), 7.44 (1H, s, CH-aromatic), 7.14 (1H, s, CH-aromatic), 4.03 (3H, s, OCH_3_), 3.98 (3H, s, OCH_3_), 3.92 (3H, s, OCH_3_), 2.54 (3H, s, CH_3_); ^13^C-NMR (150 MH_Z_, DMSO-*d_6_*) *δ*_C_: 167.53 (C=O), 157.19 (C_q_=N), 155.14 (HC=N), 152.15, 150.51, 148.81, 146.76, 142.92, 137.65, 123.15, 119.31, 114.18, 113.19, 108.02, 105.96 (4 × CH-aromatic and 8 × C_q_-aromatic ), 57.23, 56.45 and 56.18 (3 × OCH_3_), 22.47 (CH_3_); MS (EI) *m/z* (%), [M^+^ + H] 415.16 (29.06), [M^+^] 414.13 (100.00), 220.21 (49.95), 205.09 (14.82), 190.54 (4.45), 177.22 (9.00), 175.30 (2.87), 158.56 (4.71), 150.02 (22.51), 136.09 (62.14), 135.13 (38.41), 120.11 (39.52), 106.13 (25.29), 93.33 (44.22), 92.03 (22.84), 77.71 (18.43), 63.16 (65.14), 53.12 (38.90), 42.43 (32.77). Anal. Calcd. for C_19_H_18_N_4_O_7_: C, 55.07, H, 4.38, N, 13.52. Found: C, 55.26, H, 4.16, N, 13.33.

***4-[(6,7-Dimethoxy-2-methyl-4-oxo-4H-quinazolin-3-ylimino)-methyl]-benzoic acid*** (**6b**)

Off white powder (EtOH), yield (92%), m.p. 297–298 °C, ν_max_ (KBr)/cm^−1^ 3329 (OH), 3022 (CH-aromatic), 2935 (CH-aliphatic), 1736 (carboxylic C=O), 1674 (C=O), 1613 (2 × CN), 1511 and 1464 (C=C), 1397, 1377, 1331, 1251, 1211, 1169, 1130, 1108, 1018, 973, 860, 808, 769, 714, 676, 644, 607, 553, 503, 466; ^1^H-NMR (300 MH_Z_, DMSO-*d_6_*) *δ*_H_: 9.09 (1H, s, CH=N), 8.16–8.02 (4H, m, 4 × CH-Ar), 7.42 (1H, s, CH^5^-quinazolin-4(3*H*)-one), 7.11 (1H, s, CH^8^-quinazolin-4(3*H*)-one), 3.91 (3H, s, OCH_3_), 3.87 (3H, s, OCH_3_), 2.51 (3H, s, CH_3_); ^13^C-NMR (75 MH_Z_, DMSO-*d_6_*) *δ*_C_: 167.34 (carboxylic C=O), 166.76 (lactamic C=O), 156.82 (C_q_=N), 154.77 (HC=N), 151.78 and 148.41 (2 × C_q_-O), 142.44, 136.25, 134.18, 129.91, 128.74, 113.80, 107.60, 105.66 (6 × CH-aromatic and 4 × C_q_-aromatic), 56.02 and 55.74 (2 × OCH_3_), 22.03 (CH_3_) ); MS (EI) *m/z* (%) [M^+^ + H_2_] 369.00 (2.50), [M^+^] 367.24 (19.70), 220.27 (39.00), 205.15 (27.87), 177.08 (10.33), 164.24 (7.46), 147.19 (5.45), 136.17 (47.78), 105.17 (61.07), 93.14 (100.00), 91.11 (22.34), 78.05 (67.92), 65.14 (62.44), 50.13 (44.62). Anal. Calcd. for C_19_H_17_N_3_O_5_: C, 62.12, H, 4.66, N, 11.44. Found: C, 62.43, H, 4.71, N, 11.70.

***6-Chloro-3-oro-3-[(6-methoxy-naphthalen-2-ylmethylene)-amino]-2-methyl-3H-quinazolin-4-one*** (**6c**)

Beige crystals (EtOH/CHCl_3_), yield (57%), m.p. 229–230 °C, ν_max_ (KBr)/cm^−1^ 3094 and 3013 (CH-aromatic), 2965 and 2928 (CH-aliphatic), 2371, 2342, 1752, 1676 (C=O), 1598 (2 × C=N), 1476 and 1443 (C=C), 1373, 1340, 1315, 1279, 1177, 1084, 1036, 982, 991, 842, 780, 750, 702, 681, 644, 573, 544, 426, 475; ^1^H-NMR (500 MH_Z_, CDCl_3_) *δ*_H_: 9.04 (1H, s, CH=N), 8.24 (1H, d, *J* = 2.5 Hz, CH-aromatic), 8.09–8.08 (2H, m, 2 × CH-aromatic), 7.84–7.78 (2H, m, 2 × CH-aromatic), 7.68 (1H, dd, *J* = 8.5, 2.5 Hz, CH-aromatic), 7.60 (1H, d, *J* = 8.5 Hz, CH-aromatic), 7.20 (1H, app. dd, *J* = 8.8, 2.3 Hz, CH-aromatic), 7.18 (1H, d, *J* = 2.5 Hz, CH-aromatic), 3.95 (3H, s, OCH_3_), 2.67 (3H, s, CH_3_); ^13^C-NMR (125 MH_Z_, CDCl_3_) *δ*_C_: 167.25 (C=O), 159.71 (C_q_=N), 157.88 (HC=N), 154.54 (C_q_-O), 145.12, 137.25, 134.76, 132.35, 132.18, 130.65, 128.68, 128.35, 128.07, 127.83, 126.58, 123.71, 122.70, 119.82, 106.28 (9 × CH-aromatic and 6 × C_q_-aromatic), 55.54 (OCH_3_), 22.97 (CH_3_); MS (EI) *m/z* (%) [M^+^, ^37^Cl] 379.21 (2.32), [M^+^ + H, ^35^Cl] 378.17 (5.78), [M^+^, ^35^Cl] 377.11 (9.87), 336.35 (4.51), 310.32 (3.84), 285.62 (1.64), 240.77 (1.85), 203. 55 (1.92), 194.14 (10.15), 183.16 (39.46), 177.12 (2.36), 150.99 (22.32), 140.04 (27.11), 127.04 (22.47), 110.01 (51.73), 88.33 (13.20), 75.16 (100.00), 63.12 (31.44), 50.11 (10.45), 42.23 (32.65). Anal. Calcd. for C_21_H_16_ClN_3_O_2_: C, 66.76, H, 4.27, N, 11.12. Found: C, 66.95, H, 4.40, N, 11.38.

***3-[(4-Benzyloxy-3-methoxy-benzylidene)-amino]-6,7-dimethoxy-2-methyl-3H-quinazolin-4-one*** (**6d**)

Beige powder (EtOH/CHCl_3_), yield (64%), m.p.191–192 °C, ν_max_ (KBr)/cm^−1^ 3079 and 3019 (CH-aromatic), 2933 and 2868 (CH-aliphatic), 1663 (C=O), 1610 (2 × C=N), 1505, 1461 and 1423 (C=C), 1392, 1321, 1268, 1204, 1166, 1140, 1023, 919, 861, 804, 774, 747, 696, 602, 545, 433; ^1^H-NMR (500 MH_Z_, CDCl_3_) *δ*_H_: 8.98 (1H, s, CH=N), 7.76 (1H, d, *J* = 2.0 Hz, CH of 4-OC_7_H_7_,3-OCH_3_-C_6_H_3_), 7.75 (1H, s, CH^5^-quinazolin-4(3*H*)-one), 7.63 (2H, d, *J* = 7.5 Hz, 2 × CH of OC_7_H_7_), 7.56 (2H, t, *J* = 7.5 Hz, 2 × CH of OC_7_H_7_), 7.50 (1H, t, *J* = 7.5 Hz, CH of OC_7_H_7_), 7.44 (1H, app. dd, *J* = 7.5, 2.5 Hz, CH of 4-OC_7_H_7–_3-OCH_3_-C_6_H_3_), 7.24 (1H, s, CH^8^-quinazolin-4(3*H*)-one), 7.13 (1H, d, *J*= 8.0 Hz, CH of 4-OC_7_H_7–_3-OCH_3_-C_6_H_3_), 5.42 (2H, s, OCH_2_), 4.17 (3H, s, OCH_3_), 4.16 (3H, s, OCH_3_), 4.15 (3H, s, OCH_3_), 2.79 (3H, s, CH_3_); ^13^C-NMR (125 MH_Z_, CDCl_3_) *δ*_C_: 166.77 (C=O), 158.07 (C_q_=N), 154.91 (HC=N), 152.52, 152.07, 149.97 and 148.69 (4 × C_q_-O), 142.80, 136.25, 128.64, 128.07, 127.16, 125.78, 124.54, 114.59, 112.88, 109.42, 107.27, 105.95 (10 × CH-aromatic and 4 × C_q_-aromatic), 70.80 (O-CH_2_), 56.24, 56.21 and 56.02 (3 × OCH_3_), 22.57 (CH_3_); MS (EI) *m/z* (%) [M^+^ + H] 460.19 (25.82), [M^+^] 459.33 (100.00), 220.28 (2.48), 177. 36 (1.31), 121.22 (1.51), 106.13 (1.25), 92.16 (12.60), 91.26 (87.50), 90.64 (39.23), 77.15 (5.76), 65.15 (23.58), 51.09 (4.41), 42.24 (2.28), 40.97 (1.58). Anal. Calcd. for C_26_H_25_N_3_O_5_: C, 67.96, H, 5.48, N, 9.14. Found: C, 67.82, H, 5.67, N, 9.32.

***3-[(4-Benzyloxy-3-methoxy-benzylidene)-amino]-6-chloro-2-methyl-3H-quinazolin-4-one*** (**6e**)

Beige powder (EtOH/CHCl_3_), yield (81%), m.p.195–196 °C, ν_max_ (KBr)/cm^−1^ 3032 (CH-aromatic), 2932 (CH-aliphatic), 1673 (C=O), 1605 (C=N), 1574 (C=N), 1512 and 1472 and 1419 (C=C), 1378, 1318, 1275, 1205, 1139, 1083, 1036, 994, 904, 856, 834, 803, 779, 735, 691, 611, 536, 455; ^1^H-NMR (300 MH_Z_, CDCl_3_) *δ*_H_: 8.75 (1H, s, CH=N), 8.23 (1H, s, CH-aromatic), 7.74–7.22 (9H, m, 9 × CH-aromatic), 6.95 (1H, d, *J* = 8.4 Hz, CH-aromatic), 5.25 (2H, s, OCH_2_), 3.97 (3H, s, OCH_3_), 2.68 (3H, s, CH_3_); ^13^C-NMR (150 MH_Z_, CDCl_3_) *δ*_C_: 167.32 (C=O), 157.65 (C_q_=N), 154.20 (HC=N), 152.25 and 149.96 (2 × C_q_-O), 145.00, 136.15, 134.63, 132.03, 128.70, 128.56, 128.14, 127.19, 126.42, 125.40, 124.86, 122.48, 112.80, 109.34 (11 × CH-aromatic and 5 × C_q_-aromatic), 70.80 (OCH_2_), 56.06 (OCH_3_), 22.80 (CH_3_); MS (EI) *m/z* (%) [M^+^ + H, ^37^Cl] 436.12 (10.20), [M^+^, ^37^Cl] 435.12 (39.92), [M^+^ + H, ^35^Cl] 434.14 (28.06), [M^+^, ^35^Cl] 433.13 (100.00), 432.18 (21.54), 431.05 (3.46), 429.57 (2.64), 199.70 (1.21), 152.20 (1.35). Anal. Calcd. for C_24_H_20_ClN_3_O_3_: C, 66.44, H, 4.65, N, 9.68. Found: C, 66.25, H, 4.49, N, 9.45.

***6-Chloro-3-oro-3-[(5-ethyl-thiophen-2-ylmethylene)-amino]-2-methyl-3H-quinazolin-4-one*** (**6f**)

Copper crystals (MeOH/CHCl_3_), yield (67%), m.p.162–163 °C, ν_max_ (KBr)/cm^−1^ 3089 and 3013 (CH-aromatic), 2975 and 2924 CH-aliphatic), 2371, 2342, 1938, 1800, 1672 (C=O), 1601 (2 × C=N), 1472 and 1431 (C=C), 1372, 1322, 1295, 1208, 1160, 1123, 1070, 1033, 970, 949, 880, 834, 818, 782, 739, 698, 646, 572, 541, 510, 462; ^1^H-NMR (500 MH_Z_, CDCl_3_) *δ*_H_: 9.01 (1H, s, CH=N), 8.21 (1H, d, *J* = 2.5 Hz, CH^5^-quinazolin-4(3*H*)-one), 7.64 (1H, dd, *J* = 8.5, 2.5 Hz, CH^7^-quinazolin-4(3*H*)-one), 7.58 (1H, d, *J* = 8.5 Hz, CH^8^-quinazolin-4(3*H*)-one), 7.36 (1H, d, *J* = 3.5 Hz, CH^3^-thiophene), 6.86 (1H, d, *J* = 3.0 Hz, CH^4^-thiophene), 2.92 (2H, q, *J* = 7.5, Hz, CH_2__-_CH_3_), 2.62 (3H, s, CH_3_), 1.35 (3H, t, *J* = 7.5 Hz, CH_2_-CH_3_); ^13^C-NMR (150 MH_Z_, CDCl_3_) *δ*_C_: 160.22 (C=O), 157.88 (C_q_=N), 155.49 (HC=N), 154.45, 144.90, 135.26, 134.59, 134.42, 132.02, 128.54, 126.37, 124.77, 122.45 (5 × CH-aromatic and 5 × C_q_-aromatic), 24.05 (CH_2_-CH_3_), 22.77 (CH_3_), 15.57 (CH_2_-CH_3_); MS (EI) *m/z* (%) [M^+^] 331.37 (65.14), 303.05 (100.00), 195.96 (38.03), 178.35 (4.01), 151.11 (30.67), 138.76 (11.86), 110.09 (83.64), 107.50 (42.45), 95.06 (87.05), 91.09 (45.59), 76.39 (52.99), 50.33 (85.32). Anal. Calcd. for C_16_H_14_ClN_3_OS: C, 57.91, H, 4.25, N, 12.66, S, 9.66. Found, C: 57.98, H, 4.51, N, 12.89, S, 9.72.

***3-[(5-Ethyl-thiophen-2-ylmethylene)-amino]-6-fluoro-2-methyl-3H-quinazolin-4-one*** (**6g**)

Beige powder (MeOH/CHCl_3_), yield (50%), m.p.151–152 °C, ν_max_ (KBr)/cm^−1^ 3077 and 3049 (CH-aromatic), 2971 and 2927 (CH-aliphatic), 2373, 2342, 1673 (C=O), 1601 (2 × C=N), 1482 (C=C), 1380, 1345, 1316, 1272, 1237, 1186, 1125, 1097, 1037, 981, 956, 893, 814, 765, 704, 646, 595, 557, 514, 463; ^1^H-NMR (500 MH_Z_, CDCl_3_) *δ*_H_: 9.04 (1H, s, CH=N), 7.88 (1H, dd, *J* = 8.5, 3.0 Hz, CH^5^-quinazolin-4(3*H*)-one), 7.64 (1H, dd, *J* = 8.5, 5.0 Hz, CH^8^- quinazolin-4(3*H*) -one), 7.46–7.41 (2H, m, CH^7^-quinazolin-4(3*H*)-one and CH-thiophene), 6.85 (1H, d, *J* = 3.0 Hz, CH-thiophene), 2.91 (2H, q, *J* = 7.5 Hz, CH_2_-CH_3_), 2.61 (3H, s, CH_3_), 1.33 (3H, t, *J* = 7.5 Hz, CH_2_-CH_3_); ^13^C-NMR (150 MH_Z_, CDCl_3_) *δ*_C_:161.33 (C=O), 160.17 (C_q_-F), 158.18 (C_q_=N), 155.43 (HC=N), 153.46, 143.11, 135.17, 129.26, 124.76, 122.91, 122.75, 111.93, 111.77 (5 × CH-aromatic, 5 × C_q_-aromatic), 24.05 (CH_2_-CH_3_), 22.67 (CH_3_), 15.58 (CH_2_-CH_3_); MS (EI) *m/z* (%) [M^+^ + H_2_] 317.48 (3.54), [M^+^ + H] 316.11 (3.31), [M^+^] 315.16 (9.93), 286.13 (41.74), 203.61 (1.68), 178.02 (89.98), 163.06 (6.60), 150.22 (7.73), 135.13 (80.79), 122.12 (42.78), 108.07 (26.47), 94.21 (100.00), 82.15 (21.72), 75.10 (22.28), 69.10 (59.11), 57.25 (18.15), 51.15 (15.18), 45.13 (34.05). Anal. Calcd. for C_16_H_14_FN_3_OS: C, 60.94, H, 4.47, N, 13.32, S, 10.17. Found, C, 60.17, H, 4.59, N, 13.60, S, 10.25.

### 3.2. Biological Evaluation

#### 3.2.1. Antibacterial Activity

Pure standard microbial isolates collected from King Khaled University Hospital were tested in this study, including *Enterococcus faecalis* (ATCC 29122) as Gram positive bacteria and *Escherichia coli* (ATCC 25922) and *Bacteroides fragilis* (ATCC 25285) as Gram negative bacteria. Fresh cultures of each microorganism were grown on nutrient agar plates (Oxoid, UK), of which small inoculums were suspended in 5 mL nutrient broth for bacterial suspension preparation of 0.5MacFarland. Bacterial viability was investigated by estimating the colony-forming unit (CFU) ability of the bacteria incubated at different time intervals without or with appropriate amounts of the compound that were mixed with 2 × 10^7^ CFU/mL in sterile BHI broths and were incubated under shaking for 60 min at 37 °C. Samples were serially diluted into sterile BHI broths, streaked onto media agar plates, and incubated for 24 h at 37 °C. The antibacterial potency of tested compounds was expressed as the residual number of CFU with reference to the initial inoculums. Results presented as the half-maximal (50%) inhibitory concentration (IC_50_) are means of two different measurements. Ampicillin (1 mg/mL) was used as a positive standard reference.

#### 3.2.2. In Vitro Enzymatic Inhibitory Activity

##### Cyclooxygenase (COX-2) Inhibition Assay 

The compounds were resuspended in 100% DMSO at a final concentration of 10 mg/mL. Each compound was investigated in duplicates at different concentrations (50, 100, 200, and 300 µg) using the commercial COX-2 inhibitory screening assay kit (Catalog Number: 701080, Cayman Chemical Company) following the manufacturer’s instructions. Diclofenac (0.3 µg/mL) was used as the standard drug for inhibition of COX-2. The inhibitory activity was expressed as the inhibition percentage, which was determined by comparison with a control experiment. IC_50_ values were deduced from the curves.

##### Lactate Dehydrogenase-A (LDHA) Inhibition Assay

LDHA inhibition activity was investigated by measuring the amounts of consumed NADH as described by Kim [67] and his group. Briefly, different concentrations of each compound (50, 100, 200, and 300 µg) were incubated in buffer containing 20 mM of HEPES-K+ (pH 7.2), 20 μM of NADH, 2 mM of pyruvate, and 10 ng of purified recombinant human LDHA protein for 10 min. The fluorescence of NADH, which has an excitation wavelength of 340 nm and emission wavelength of 460 nm, was detected using a Spectrofluorometer. Oxamate (88 µg/mL) was used as a standard inhibitor of LDHA.

#### 3.2.3. In Vitro Cytotoxicity Assay

Cytotoxic potency was examined on human colon cancer cell lines HCT-116 and LoVo (American Type Culture Collection, Manassas, VA, USA) using various amounts of tested compounds (50, 100, 200, and 400 µg). Samples were diluted in Dulbecco’s modified Eagles medium, consisting of 10% fetal bovine serum, added to cells grown and cultured for 24 h in a 5% CO_2_-humidified incubator at 37 °C. Then, the activity of lactate dehydrogenase released from damaged cells was determined in the collected supernatant aliquots using an ELISA end-point assay (Benchmark Plus, Bio-Rad, CA, USA). The 0.1% Triton X-100 in the assay medium and the assay medium only were used as positive and negative controls, respectively. Cell viability, expressed as a relative percentage of the OD values (at 550 nm) for compound-treated cells (final concentration of 200 µg) and the control, is shown as mean ± SD (*n* = 2). The plot of the cell viability (%) versus the compound concentration was also performed to determine the compound concentration providing 50% inhibition (IC_50_).

### 3.3. Molecular Docking

The structures were drawn using ChemSketch freeware (www.acdlabs.com) followed by their optimization using MMFF94 force field using Avogadro (ver. 1.2) using default settings. The structures of the target proteins were retrieved from Protein Data Bank: www.rcsb.org (accessed on 8 January 2021). For COX-2, the pdb 3NT1 was selected on the basis of X-ray resolution and sequence. For *Bacteroides fragilis* and *E. coli*, the pdb: 1A8T and 4XVE were selected, respectively, on the basis of X-ray resolution and due to the presence of an inhibitor possessing quinazoline ring. The presence of an inhibitor possessing a similar ring helped to identify the appropriate enzyme and the active site of the enzyme. Then, the structures of the proteins were curated using UCSF Chimera: http://www.cgl.ucsf.edu/chimera/ (accessed on 8 January 2021). Molecular docking was performed using NRG Suite, which is a python and C++ based PyMOL plugin. It identifies the surface cavities in a protein using FlexAID and treat them as target binding-sites for docking simulations. In the present work, following default settings were used to get optimum performance of NRGsuite: binding sites input method, spherical shape, spacing of three dimensional grid: 0.375 Å, side chain flexibility—no, ligand flexibility—yes, ligand pose as reference—yes, constraints—no, HET groups—included water molecules, van der Walls permeability—0.1, solvent types—no type, number of chromosomes—1000, number of generations—1000, fitness model—share, reproduction model—population boom, and number of TOP complexes—10.

### 3.4. ADMET Predictions

The ADMET were predicted using pK-CSM [37]. For this, the SMILES notations of the molecules were uploaded on the web-server of pk-CSM: http://biosig.unimelb.edu.au/pkcsm/prediction (accessed on 1 March 2021). The server also provided analysis of molecules as per Lipinski’s rule of five.

## 4. Conclusions

Herein, the synthesis, characterization and multiple biological evaluations of a new series of Schiff’s bases incorporating quinoxalin-2(1*H*)one and 3*H*-quinazolin-4-one scaffolds are described. The results, indicated that compounds (**4c**) and (**6b**) would be beneficial for CRC prevention due to their broad spectrum antibacterial activity against *E. feacalis*, *B. fragilis,* and *E. coil*. Moreover, aldimines (**6e**, **6d**, and **6a**) and (**6g**, **6a**, and **4d**) showed the strong enzymatic inhibition against COX-2 and LDHA, respectively. Cytotoxicity assays against HCT-116 and LoVo cells revealed that the two cell lines were very sensitive to compound (**6d**), which reduced the percentage of viable cells to 18.50% and 26.75% with IC_50_ values of 100.0 and 127.5 µg/mL, respectively. The bioactive compounds: (**6d**; anti-COX-2), (**4c**; anti-*B. fragilis*) and (**6a**; anti-*E. coli*) were subjected to in silico molecular docking analyses in the active sites of COX-2, metallo-β-lactamase, and 17-beta-HSD5 enzymes, respectively. These analyses were useful for identifying the important structural features of the present compounds responsible for the good interactions with the target enzymes and thus, they should be retained in future modifications. Finally, the ADMET characteristics of all synthesized compounds were predicted and revealed that the promising anti-CRC candidate (**6d**) possesses desirable pharmacokinetic properties positioning it as a promising lead for further optimization.

## Data Availability

The data presented in this study are available in the experimental section or in the Supplementary Material.

## Supplementary Materials

The following are available online. The supplementary data include Tables S1–S10 and Figures S1–S3.

## Sample Availability

Samples of the compounds are available from the authors.
