# Supplementary material for: Synthesis and Characterization of Some New Quinoxalin-2(1H)one and 2-Methyl-3H-quinazolin-4-one Derivatives Targeting the Onset and Progression of CRC with SAR, Molecular Docking, and ADMET Analyses
Source: Molecules. 2021 May 23;26(11):3121. doi: 10.3390/molecules26113121 (PMC8197120; doi:10.3390/molecules26113121)
Supplement: Supplementary file 1 [file molecules-26-03121-s001.zip › molecules-1215150-supplementary.pdf]

Synthesis and Characterization of Some New Quinoxalin-2(*1H*)one and 2-Methyl-3*H*-quinazolin-4-one Derivatives Targeting the Onset and Progression of CRC with SAR, Molecular Docking, and ADMET Analyses

Nahed N. E. El-Sayed, Taghreed M. Al-Otaibi, Mona Alonazi, Vijay H. Masand, Assem Barakat, Zainab M. Almarhoon, Abir Ben Bacha

Supplementary materials

Table S1. Evaluation of the antibacterial activity of the synthesized quinoxaline and quinazoline derivatives by determination of the concentration required for 50% inhibition (IC_{50} µg/ml) values. Results are the mean values of two separate determinations ± standard deviation (SD).

Compound #	*Bacteroides fragilis* (ATCC 25285)	*Enterococcus faecalis* (ATCC 29122)	*Escherichia coli* (ATCC 25922)
	**Mean IC_{50} ± SD µg/ml**	**Mean IC_{50} ± SD µg/ml**	**Mean IC_{50} ± SD µg/ml**
**1**	19.35 ± 1.484924	20.25 ±1.06066	25.85 ± 0.77817
**2**	14.50 ± 0.989949	13.60 ± 0.565685	13.65 ± 1.343503
**4a**	13.30 ± 0.848528	27.00 ± 2.828427	15.00 ± 0.565685
**4b**	40.65 ± 2.333452	30.30 ± 2.404163	13.60 ± 0.848528
**4c**	8.05 ± 0.777817	9.60 ± 0.848528	12.65 ± 0.777817
**4d**	28.25 ± 1.767767	10.80 ± 1.131371	30.55 ± 2.616295
**4e**	27.10 ± 1.838478	18.20 ± 0.989949	31.45 ± 1.484924
**5b**	37.00 ± 2.828427	27.50 ± 2.12132	13.55 ± 1.484924
**6a**	10.90 ± 0.565685	13.85 ± 0.494975	7.90 ± 0.848528
**6b**	10.35 ± 1.202082	12.65 ± 0.919239	18.20 ± 0.989949
**6c**	30.05 ± 0.636396	20.45 ± 1.202082	26.90 ± 1.555635
**6d**	9.00 ± 0.989949	15.15 ± 0.919239	18.40 ± 1.272792
**6e**	12.75 ± 0.919239	12.40 ±0.848528	15.65 ± 0.919239
**6f**	27.90 ± 1.555635	22.55 ± 0.636396	33.40 ± 3.394113
**6g**	19.90 ± 2.262742	17.25 ±1.767767	24.75 ± 1.767767
**Ampicillin**	16.15 ± 1.202082	12.75 ± 1.06066	20.25 ± 1.767767

Table S2. Evaluation of the cyclooxygenase-2 (COX-2) inhibitory properties of the synthesized quinoxaline and quinazoline derivatives. Results are the mean values of two separate determinations ± standard deviation (SD).

Compound #	Mean Inhibition % at 100 μg/ml ± SD	Mean Inhibition % at 200 μg/ml ± SD
**1**	30.85 ±2.616295	48.10 ±2.969848
**2**	7.55 ± 0.777817	15.45 ±2.192031
**4a**	4.65 ± 0.494975	12.05 ±2.05061
**4b**	12.35 ± 0.919239	21.05 ±2.899138
**4c**	36.45 ± 3.464823	61.10 ±1.979899
**4d**	14.45 ± 0.777817	28.90 ±2.12132
**4e**	30.30 ± 1.838478	54.00 ±2.262742
**5b**	5.70 ± 0.989949	9.05 ±1.484924
**6a**	42.90 ± 2.969848	82.95 ±2.474874
**6b**	37.10 ± 2.687006	68.95 ±2.757716
**6c**	23.80 ± 2.545584	36.85 ±1.626346
**6d**	50.20 ± 3.11127	97.45 ±1.06066
**6e**	57.85 ± 4.030509	100.00 ±0.00000
**6f**	10.00 ±1.414214	20.70 ±2.404163
**6g**	28.75 ±2.474874	48.65 ±3.747666
**Diclofenac (0.3 μg/ml)**	100 ± 0.00	

Table S3. Evaluation of the LDHA inhibitory properties of the synthesized quinoxaline and quinazoline derivatives. Results are the mean values of two separate determinations ± standard deviation (SD).

Compound #	Mean Inhibition % at 100 μg/ml ± SD	Mean Inhibition % at 200 μg/ml ± SD
**1**	33.8 ± 2.545584	68.20 ± 2.545584
**2**	26.7 ± 1.838478	47.15 ± 1.626346
**4a**	20.3 ± 1.626346	28.25 ± 2.474874
**4b**	27.4 ± 2.404163	35.40 ± 3.818377
**4c**	40.65 ± 2.333452	64.20 ± 6.22254
**4d**	47.40 ± 3.959798	84.95 ± 4.17193
**4e**	21.05 ± 2.192031	31.20 ± 3.11127
**5b**	11.75 ± 1.06066	21.20 ± 2.404163
**6a**	62.55 ± 4.030509	89.70 ± 2.969848
**6b**	32.00 ± 2.545584	49.25 ± 1.06066
**6c**	13.35 ± 0.919239	23.00 ± 2.828427
**6d**	31.25 ± 1.626346	44.40 ± 3.394113
**6e**	44.75 ± 2.899138	76.85 ± 1.767767
**6f**	27.05 ± 1.484924	40.05 ± 2.757716
**6g**	53.60 ± 3.252691	89.85 ± 0.919239
**Oxamate (88 μg/ml)**	100 .00 ± 0.00	

Table S4. Cytotoxic effects of some bioactive Schiff's bases on LoVo and HCT-116 colorectal cells. Cell viability is expressed as a relative percentage of the optical density (OD) values determined at 600 nm in compound-treated cells (200 µg/ml) and the control. Results are the mean values of two separate determinations ± standard deviation (SD).

Compound #	Mean % Cell Viability ± SD<br>CRC-LoVo cells	Mean %Cell Viability ± SD<br>CRC-HCT-116 cells
**4c**	72.15 ± 4.454773	77.3 ± 3.252691
**4d**	60.00 ± 4.242641	65.5 ± 3.535534
**6a**	92.15 ± 2.616295	91.2 ± 3.11127
**6b**	54.25 ± 2.474874	47.0 ± 2.828427
**6d**	26.75 ± 2.474874	18.5± 2.12132
**6e**	93.40 ± 4.808326	89.0 ± 2.828427
**6g**	88.15 ± 2.616295	86.5 ± 2.12132
**Negative Control Assay medium**	99.50 ± 0.707107	100.0 ± 0.00000
**Positive Control Triton X-100 (0.1%)**	0.00	

Table S5. Compliance of the synthesized compounds to Lipinski's rule of five.

compound	Molecular Weight	LogP	# Rotatable Bonds	# HB Acceptors	# HB Donors	Surface Area	# Ro5 violation	Ro5 violation
**1**	162.148	0.2164	0	2	2	66.875	0	NO
**2**	174.163	-0.2594	0	2	2	72.919	0	NO
**4a**	344.758	2.1586	3	5	3	140.473	0	NO
**4b**	436.209	2.1098	3	5	3	149.431	0	NO
**4c**	355.31	1.4134	4	7	3	144.822	1	Yes
**4d**	308.297	1.4892	3	4	3	129.217	0	NO
**4e**	344.374	2.9528	3	4	2	148.057	0	NO
**5b**	209.636	1.07212	0	4	1	84.902	0	NO
**6a**	414.374	2.22672	6	10	1	169.459	2	Yes
**6b**	367.361	2.30252	5	7	1	153.854	2	Yes
**6c**	377.831	4.40232	3	5	0	160.04	1	Yes
**6d**	459.502	4.19192	8	8	0	196.547	2	Yes
**6e**	433.895	4.82812	6	6	0	183.894	2	Yes
**6f**	331.828	3.86442	3	5	0	136.25	0	NO
**6g**	315.373	3.35012	3	5	0	130.112	0	NO

Overall molecular weight range 162.148 – 459.502, LogP range −0.2594 to 4.40232, # HB Acceptors range 2–10, # HB Donors range 0–3, Surface Area range 66.875 – 196.547, and # Rotatable Bonds range 0 – 8.

Table S6. Prediction of the absorption parameters for the synthesized compounds.

Property	Absorption						
Parameter	H_2O Sol.	Caco2 permeability	Intestinal absorption (human)	Skin Permeability	P-gp substrat	P-gp I inhibitor	P-gp II inhibitor
Unit	Numeric (log mol/L)	Numeric (log Papp in 10^{-6} cm/s)	Numeric (%absorbed)	Numeric (log Kp)	Categorical (Yes/No)	Categorical (Yes/No)	Categorical (Yes/No)
**Comp. #**							
**1**	-2.287	0.646	79.177	-3.039	Yes	No	No
**2**	-2.486	0.699	76.48	-3.054	Yes	No	No
**4a**	-3.348	0.513	79.275	-2.866	Yes	No	No
**4b**	-3.379	0.533	77.173	-2.865	Yes	No	No
**4c**	-3.127	-0.108	75.33	-2.741	Yes	No	No
**4d**	-3.472	-0.245	59.924	-2.726	Yes	No	No
**4e**	-4.316	0.921	94.196	-2.762	Yes	No	Yes
**5b**	-2.683	1.16	89.231	-3.018	Yes	No	No
**6a**	-4.336	-0.003	88.424	-2.746	Yes	Yes	No
**6b**	-4.498	0.987	73.007	-2.619	No	No	No
**6c**	-5.178	1.138	97.429	-2.669	No	No	Yes
**6d**	-5.21	1.314	99.584	-2.735	No	Yes	Yes
**6e**	-5.419	1.123	96.899	-2.731	No	Yes	Yes
**6f**	-5.042	1.42	94.978	-2.414	No	No	No
**6g**	-4.58	1.388	95.881	-2.433	No	No	No

H_2O Sol.: water solubility; P-gp= P-glycoprotein; Caco2 permeability refers to intestinal permeability in *vitro* which is assayed by determination of permeability coefficients across monolayers of the human colon carcinoma cell line Caco-2 model.

Table S7. Prediction of the distribution parameters for the synthesized compounds.

Property	Distribution			
Comp.	VDss[a] (human)	Fraction unbound (human)	BBB permeability	CNS permeability
Unit	Numeric (log L/kg)	Numeric (Fu)	Numeric (log BB)	Numeric (log PS)
**1**	-0.301	0.493	-0.357	-2.414
**2**	-0.313	0.488	-0.109	-2.505
**4a**	0.023	0.188	-1.023	-2.314
**4b**	0.053	0.186	-1.03	-2.324
**4c**	-0.337	0.145	-1.078	-2.587
**4d**	-1.368	0.163	-0.932	-2.398
**4e**	-0.262	0.03	-0.083	-1.914
**5b**	-0.087	0.377	-0.274	-2.845
**6a**	-0.433	0.115	-1.399	-3.304
**6b**	-1.153	0.16	-1.026	-3.143
**6c**	0.148	0.182	0.312	-1.706
**6d**	-0.387	0.103	-1.1	-3.046
**6e**	-0.173	0.111	0.067	-1.992
**6f**	0.082	0.098	0.338	-1.368
**6g**	-0.088	0.119	0.331	-1.394

[a]**VDss:** volume of distribution at steady state; FU; fraction unit; BBB: blood brain barrier; log BB: the logarithm of the ratio of steady-state concentration of drug in brain to that in blood; CNS: central nervous system; log PS: logarithm of permeability surface-area.

Table S8. Prediction of the metabolism parameters for the synthesized compounds.

Property	Metabolism						
**Comp.**	**CYP2D6 substrate**	**CYP3A4 substrate**	**CYP1A2 inhibitor**	**CYP2C19 inhibitor**	**CYP2C9 inhibitor**	**CYP2D6 inhibitor**	**CYP3A4 inhibitor**
**Unit**	Categorical (Yes/No)	Categorical (Yes/No)	Categorical (Yes/No)	Categorical (Yes/No)	Categorical (Yes/No)	Categorical (Yes/No)	Categorical (Yes/No)
**1**	No	No	Yes	No	No	No	No
**2**	No	No	Yes	No	No	No	No
**4a**	No	Yes	Yes	No	No	No	No
**4b**	No	Yes	Yes	No	Yes	No	No
**4c**	No	Yes	Yes	No	No	No	No
**4d**	Yes	No	Yes	No	No	No	No
**4e**	No	Yes	Yes	Yes	Yes	No	No
**5b**	No	No	Yes	No	No	No	No
**6a**	No	Yes	No	No	No	No	Yes
**6b**	No	No	No	No	No	No	No
**6c**	No	Yes	Yes	Yes	Yes	No	Yes
**6d**	No	Yes	No	Yes	Yes	No	Yes
**6e**	No	Yes	Yes	Yes	Yes	No	Yes
**6f**	No	Yes	Yes	Yes	Yes	No	Yes
**6g**	No	Yes	Yes	Yes	Yes	No	Yes

Table S9. Prediction of the excretion parameters for the synthesized compounds.

Property	Excretion	
**Comp.**	**Total Clearance**	**Renal OCT2[a] substrate**
**Unit**	Numeric (log ml/min/kg)	Categorical (Yes/No)
**1**	0.395	No
**2**	0.29	No
**4a**	0.324	No
**4b**	-0.063	No
**4c**	0.379	No
**4d**	0.591	No
**4e**	0.273	No
**5b**	-0.002	No
**6a**	0.837	No
**6b**	0.678	No
**6c**	0.064	No
**6d**	0.627	No
**6e**	0.03	No
**6f**	0.05	Yes
**6g**	0.032	Yes

[a] OCT2: Organic cation transporter 2.

Table S10. Prediction of the toxicity parameters for the synthesized compounds.

Property	Toxicity									
**Comp.**	**AMES toxicity**	**Max. tolerated dose (human)**	**hERG I inhibitor**	**hERG II inhibitor**	**Oral Rat Acute Toxicity (LD50)**	**Oral Rat Chronic Toxicity (LOAEL)**	**Hepato-toxicity**	**Skin Sensitization**	**T. Pyriformis toxicity**	**Minnow toxicity**
**Unit**	Categorical (Yes/No)	Numeric (log mg/kg/day)	Categorical (Yes/No)	Categorical (Yes/No)	Numeric (mol/kg)	Numeric (log mg/kg_bw/day)	Categorical (Yes/No)	Categorical (Yes/No)	Numeric (log µg/L)	Numeric (log mM)
**1**	No	-0.208	No	No	1.999	1.171	No	No	0.24	1.761
**2**	Yes	-0.232	No	No	2.019	1.806	Yes	No	0.251	1.761
**4a**	No	0.122	No	Yes	2.021	2.252	Yes	No	0.56	1.398
**4b**	No	0.104	No	Yes	2.028		Yes	No	0.556	1.112
**4c**	No	0.242	No	No	3.203	2.901	Yes	No	0.355	2.472
**4d**	No	0.819	No	No	2.515	2.039	No	No	0.299	1.986
**4e**	Yes	-0.041	No	Yes	2.431	1.357	Yes	No	0.427	1.821
**5b**	Yes	0.383	No	No	2.086	1.487	No	No	0.621	1.664
**6a**	No	0.241	No	Yes	2.028	1.346	Yes	No	0.305	0.323
**6b**	No	0.743	No	No	2.915	1.433	Yes	No	0.293	0.78
**6c**	Yes	0.346	No	Yes	2.587	0.964	Yes	No	0.36	-2.47
**6d**	No	0.564	No	Yes	2.711	1.077	No	No	0.293	-1.505
**6e**	No	0.783	No	Yes	2.684	1.102	Yes	No	0.304	-1.737
**6f**	No	0.152	No	No	2.202	1.815	Yes	No	1.304	-0.545
**6g**	No	0.135	No	No	2.111	1.966	Yes	No	1.215	-0.212

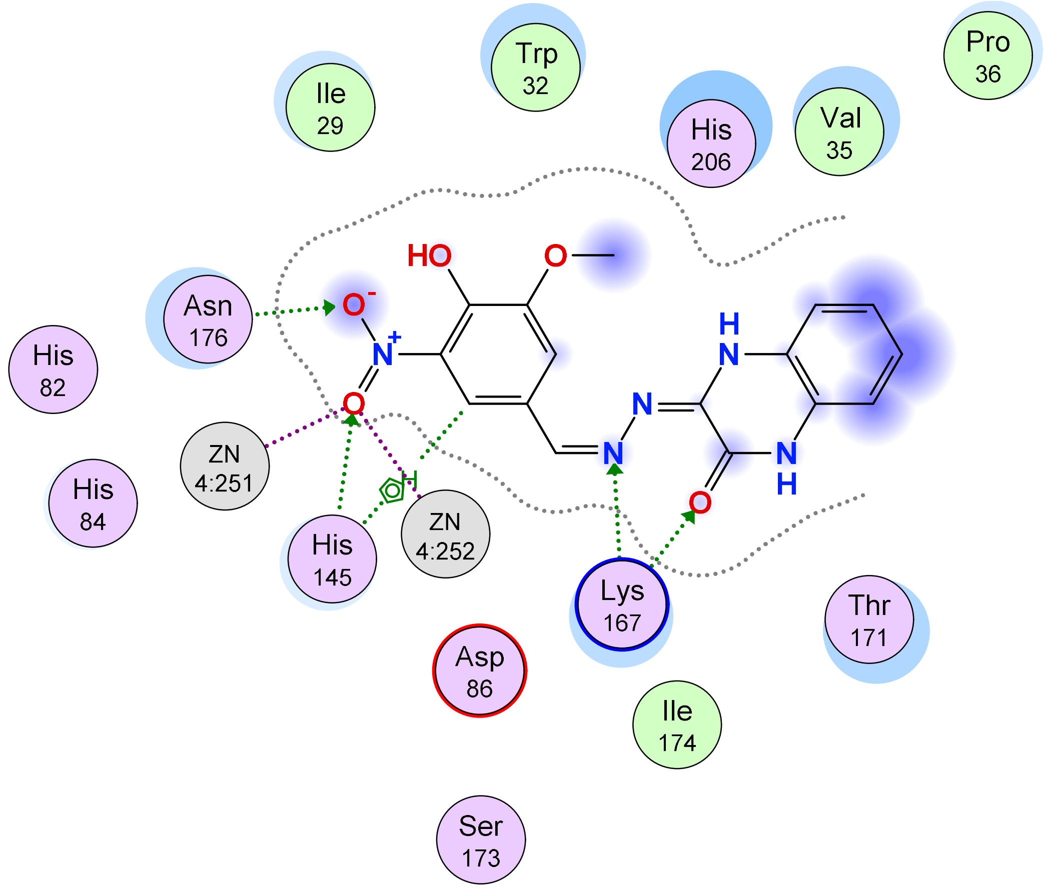

Figure S1. 2D docking pose of molecule (**4c**) in the active site of *β*-lactamase pdb: 1a8t.

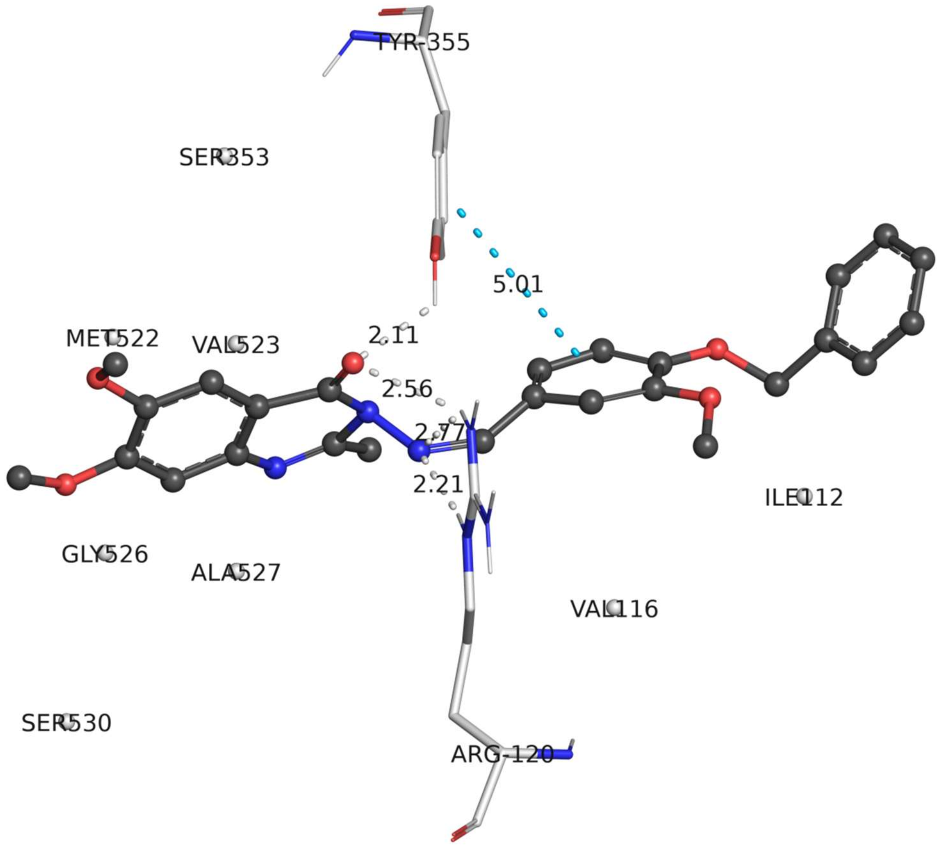

Figure S2. Docking pose of compound **(6d**) within active site of COX-2.

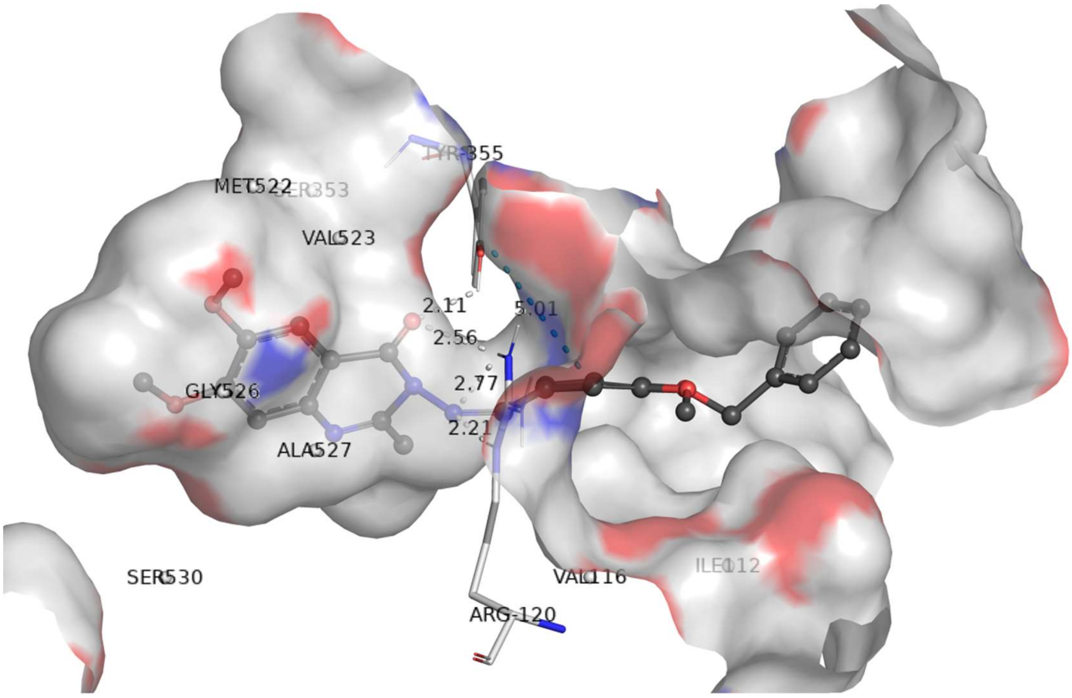

Figure S3. Docking pose of compound (6d) within the active pocket of COX-2.
